# Machine learning approaches for predicting preventive maintenance costs of expressways in Xinjiang

**DOI:** 10.1371/journal.pone.0349595

**Published:** 2026-06-16

**Authors:** Chunyan Jia, Jianhui An, Shengqiang Ma, Xiaomin Dai

**Affiliations:** 1 School of Architecture and Engineering, Xinjiang University, Urumqi, China; 2 Xinjiang Jiaotou Maintenance Group Co., Ltd., Urumqi, China; 3 Xinjiang Jiaotou Engineering Technology Development Co., Ltd., Urumqi, China; 4 School of Traffic and Transportation Engineering, Xinjiang University, Urumqi, China; 5 Xinjiang Key Laboratory of Green Construction and Maintenance of Transportation Infrastructure and Intelligent Traffic Control, Urumqi, China; Sunway University, MALAYSIA

## Abstract

With the expansion of high-speed road mileage and the aging of road networks, maintenance workloads have increased dramatically. Accurately forecasting preventive maintenance costs is a practical approach to rationally control maintenance expenditure, enhance capital utilization efficiency, and ensure the long-term stable operation of the road network. However, the current predictive model for preventive maintenance costs fails to adequately account for variations in types of preventive maintenance measures, limiting their ability to capture the actual cost variation patterns under different engineering conditions. Consequently, there remains scope for improvement in prediction accuracy. In this study, we developed three meta-heuristic-optimized hybrid models, each tailored to a specific asphalt pavement preventive maintenance measure: 1) the fruit fly optimization algorithm (FOA)-enhanced XGBoost model for crack filling; 2) the hiking optimization algorithm (HOA)-enhanced RF model for surface sealing; 3) the particle swarm optimization (PSO)-enhanced BPNN model for overlay. A case study demonstrates that FOA-XGBoost (R² = 0.8622, MAE = 0.0552) improved XGBoost’s R² by 0.0526.

In contrast, HOA-RF (MSE = 0.0032, RMSE = 0.0565) outperformed RF with lower error metrics. Furthermore, PSO-BPNN achieved the highest R² (0.9277) and the lowest MAE (0.0419) compared to BPNN. All models maintained MAPE below 5%. To further support the reliability of the results, the Wilcoxon signed-rank test was conducted to assess the statistical significance of model performance differences. In addition, nested cross-validation and sensitivity analysis were performed to evaluate the robustness and stability of the proposed models. These findings indicate that the optimized hybrid model has demonstrated improvements in both predictive accuracy and stability. Consequently, the developed models may provide useful support for maintenance cost estimation and resource allocation in expressway maintenance management.

## 1. Introduction

Expressways constitute the fundamental framework of the global transportation system and serve as a critical foundation for economic growth. The Ministry of Transport has stated that China’s expressway network spanned 191,000 kilometers as of July 2025. Owing to the cumulative effects exerted by diverse environmental conditions and prolonged traffic loads, asphalt pavements commonly develop defects such as cracks, ruts, and deformation. These issues not only compromise driving safety and comfort but also substantially affect the overall operational efficiency of the road infrastructure [1–3]. Preventive maintenance is a key means of mitigating road deterioration and extending road service life. Maintaining roads in good condition can minimize disruption to traffic and long-term expenditure, which is consistent with the infrastructure-focused sustainable development goals established by the United Nations [4–6].

China has entered the initial phase of peak construction and comprehensive maintenance of its road network, with funding for pavement maintenance increasing consistently to an average annual amount of $6.19 billion [7]. The Pavement Management System (PMS) is a core tool for modern road asset management, primarily used for the full life cycle management of pavement facilities such as expressways and urban roads. The cost module in the PMS system is used to optimize resource allocation; therefore, accurately forecasting pavement preventive maintenance expenditures is a critical component of management decision-making, with the precision of predictions directly influencing the efficiency of fund utilization. Constructing scientific prediction models can provide quantitative support for the formulation of maintenance plans and the dynamic allocation of funds, ultimately achieving optimized control of maintenance costs and a coordinated improvement in pavement service performance [8].

In recent years, the consistent growth of expressway mileage has been accompanied by a notable increase in the probability of premature pavement deterioration. As a result, numerous pavement performance prediction models have been developed by many researchers for this purpose. The majority of existing research focuses on forecasting routine maintenance costs, while there is a relative scarcity of studies addressing the prediction of preventive maintenance costs [9–11]. Conventional statistical approaches are extensively employed in forecasting future costs, including the Ordinary Least Squares methods (OLS) [12], Multiple regression analysis (MRA) [13], and Generalized linear model (GLM) [14]. These models typically exhibit limitations in accurately and stably predicting outcomes because they are unable to effectively manage complex nonlinear relationships, capture interactions among multiple variables, and adapt to intricate data structures.

To address the above issues, artificial intelligence (AI)-driven machine learning models have been extensively applied to cost prediction across fields including construction, finance, and healthcare [15–17]. It includes not only deep learning algorithms such as fully connected artificial neural networks (ANN), convolutional neural networks (CNN), and backpropagation neural networks (BPNN), but also conventional machine learning algorithms such as extreme gradient boosting (XGBoost), random forests (RF), and support vector machines (SVM). These machine learning algorithms can capture nonlinear relationships within data, thereby enhancing their predictive performance in real-world applications. For example, Wang et al. [18] used ANN, CNN, and regression models to predict bridge maintenance costs. The results showed that the CNN model performed best among the three models, demonstrating superior prediction accuracy. Xue et al. [19] developed CNN model for predicting highway construction costs, incorporating 25 input parameters that include province, mileage, administrative hierarchy, design speed, and traffic volume, among others. Notably, this parameter set encompasses 10 innovative factors related explicitly to bridges and tunnels. The model achieved a mean absolute percentage error (MAPE) of 17% in its cost prediction performance. Wu et al. [20] employed both the BPNN model and the SVM model to predict the total hospitalization costs for patients with bronchopneumonia. Similarly, Alshboul et al. [21] applied three machine learning algorithms—XGBOOST, Deep Neural Network (DNN), and RF—to estimate the costs associated with green building projects. Among these models, XGBoost has shown superior performance in prediction, achieving an R² value of 0.96, thereby highlighting its effectiveness in green building cost prediction tasks.

However, with the advancement of computer technology, the randomness in hyperparameter selection in individual machine learning models has introduced challenges to the accuracy and stability of model predictions [22–24]. Conventional parameter tuning approaches predominantly depend on manual adjustments, which are not only inefficient but also exhibit limited adaptability to complex model architectures [25,26]. Consequently, a growing body of research has focused on integrating metaheuristic algorithms with machine learning models to develop more effective hybrid models [27,28]. Notable examples of such algorithms include Particle Swarm Optimization (PSO), Genetic Algorithm (GA), Fruit Fly Optimization Algorithm (FOA), and Hiking Optimization Algorithm (HOA), etc. For instance, Wang et al. [29] utilized a BPNN optimized by PSO to predict the self-ignition risk of extraction boreholes. The prediction performance of this model outperformed that of the standalone BPNN, as well as BPNN models optimized by GA, Sparrow Search Algorithm (SSA), and Marine Predators Algorithm (MPA). Uzunoglu et al. [30] employed a learning-to-rank machine learning model to select the optimal initial solution algorithm from 17 heuristic algorithms and subsequently improved the initial solution using a Genetic Algorithm. Their results demonstrated that the Genetic Algorithm yielded the best performance across most instances. Li et al. [31] combined the FOA with CatBoost to develop the FOA-CatBoost model for predicting the sintering properties of iron ore powders. This model outperformed the PSO-CatBoost, FOA-XGBoost, and unoptimized CatBoost models in terms of predictive performance. Samiei Moghaddam et al. [32] introduced a multi-objective optimization framework based on the HOA, comparing it with six benchmark algorithms, including Komodo Dragon Optimization Algorithm (KMA), PSO, and GA. The results showed that the HOA model reduced operational costs by 19.3% and energy losses by 59.7%.

From the above analysis, it is evident that integrating meta-heuristic algorithms with machine learning models has become a prevailing trend, owing to their capability to reduce errors and enhance predictive performance. Nevertheless, the application of such hybrid models in the context of expressways’ preventive maintenance costs prediction remains relatively limited. In this study, preventive maintenance activities are categorized based on specific treatment types, and hybrid models are developed accordingly for cost prediction purposes.

The contributions of this study are as follows:

1. To address the limitation of single models failing to accommodate diverse maintenance techniques in current road maintenance cost forecasting, this study proposes a classification modeling approach grounded in engineering principles. According to the treatment objectives for different pavement distresses, preventive maintenance measures are divided into crack filling, surface sealing, and overlay. For each of these three measures, XGBoost, RF, and BPNN models were established. This approach enables precise matching between prediction models and the attributes of maintenance techniques, effectively overcoming the issue of homogeneous modeling in traditional methods.
2. This study introduces meta-heuristic algorithms, including FOA, HOA, and PSO to optimize the hyperparameters of three base prediction models: XGBoost, RF, and BPNN model. It addresses the issues of machine learning models easily falling into local optima and being sensitive to parameter selection in complex, nonlinear scenarios. Through a comparison of the model’s performance before and after optimization, showing improved predictive performance of the meta-heuristic-optimized hybrid models.
3. A comprehensive technical framework encompassing the entire process has been established, including data preprocessing, feature engineering, model construction, hyperparameter optimization, and performance evaluation. Furthermore, a nested cross-validation framework was implemented to assess the robustness and generalization ability of model performance, while non-parametric statistical testing and sensitivity analysis were conducted to provide additional support for the reliability of model comparisons and to offer insights into the influencing factors of maintenance costs within the scope of this study.

The rest of this paper is organized as follows: Section 2 presents the methodologies and model evaluation metrics. Section 3 presents the case study. Finally, Section 4 summarizes the research findings and offers some suggestions for future research.

## 2. Materials and methods

**Fig 1:**
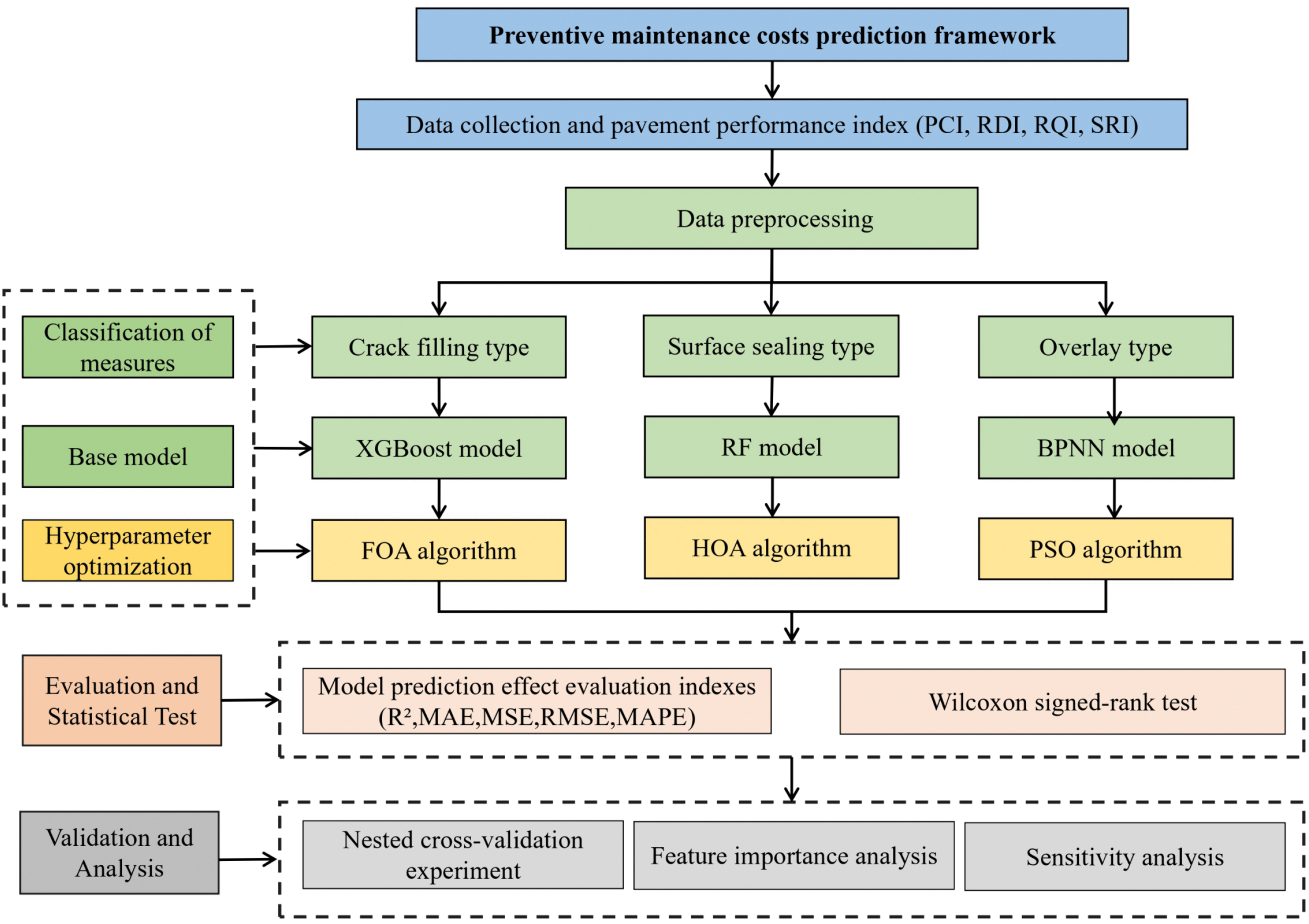
The comprehensive framework for the cost-prediction system.

This study develops a comprehensive framework for predicting preventive maintenance costs of expressways. The comprehensive framework for the cost-prediction system is depicted in Fig 1. Based on the principles of maintenance technology and engineering practice, preventive maintenance measures are systematically divided into three major technical systems: crack filling, surface sealing, and overlay. For each of these three preventive maintenance techniques, XGBoost, RF, and BPNN models have been developed. Additionally, to enhance the models’ prediction accuracy, robustness, and generalizability, meta-heuristic algorithms were utilized for hyperparameter optimization, resulting in three hybrid models: FOA-XGBoost, HOA-RF, and PSO-BPNN.

### 2.1. Pavement distress detection and technical condition indicators

**Fig 2:**
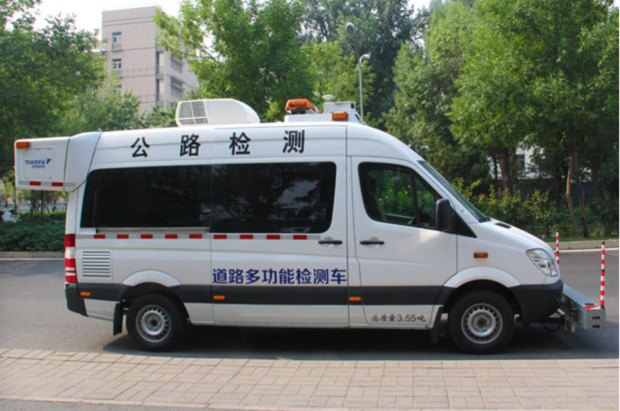
Road condition inspection vehicle.

**Fig 3:**
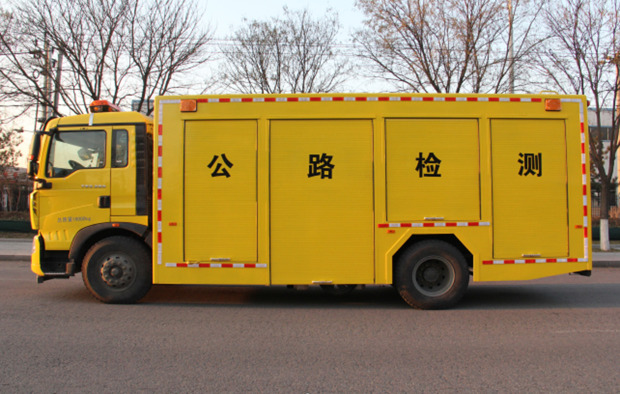
Road surface lateral force coefficient test vehicle.

As specified in “China Highway Technical Condition Assessment Standard JTG 5210–2018”, the indicators reflecting pavement technical conditions include the Pavement Condition Index (PCI), Road Quality Index (RQI), Road Rutting Depth Index (RDI), and Skid Resistance Index (SRI), as shown in the calculations in Table 1. Meanwhile, maintenance workloads are recognized as a critical factor affecting the allocation of road maintenance resources and the management of costs. Unlike the visual assessment of road surface conditions, maintenance workloads cannot be accurately estimated through visual inspection alone. Road condition inspection vehicles use automated equipment to collect road surface images, three-dimensional textures, and laser data, and collect data on the number of surface defects such as cracks, potholes, and ruts. These data are then integrated with dynamic performance indicators such as smoothness and skid resistance to produce comprehensive evaluation outcomes. Fig 2 and Fig 3 show the working scenes of integrated road surface inspection equipment.

**Table 1:** Road surface condition index.

Index	Calculation method	Parameters
PCI	$\begin{array}{cc} PCI & =100-15DR^{0.412}\\ DR & =100\times \frac{\sum_{i=1}^{21}w_{i}A_{i}}{A} \end{array}$	*DR* is the pavement damage rate (%); *w*_*i*_ is the weighting factor or conversion coefficient for pavement distress type *i*; *A*_*i*_ is the accumulated area of pavement distress type *i* (m^2^); *A* is the total surveyed pavement area (m)
RQI	$RQI=\frac{100}{1+0.026e^{0.65IRI}}$	*IRI* is the International roughness Index (m/km)
RDI	$RDI=\left\{ \begin{array}{cc} 100-RD & \left(RD\leq 10\right)\\ 90-3(RD-10) & \left(10<RD\leq 40\right)\\ 0 & \left(RD>40\right) \end{array}\right.$	*RD* is the rut depth (mm)
SRI	$SRI=\frac{65}{1+28.6e^{-0.105SFC}}+35$	*SFC* is the side force coefficient

### 2.2. Data sources and preprocessing

This study collected data from preventive maintenance projects implemented on 13 expressways in Xinjiang, China (G7, G30, G3003, G3012, G3013, G3014, G3015, G3016, S11, S12, S13, S16, and S22) over the period from 2021 to 2025. The dataset comprises 239 independent observations derived from actual engineering records, which are shown in S1 Table. Each observation corresponds to a specific preventive maintenance project conducted on a defined expressway section within a given year, and can be formally represented as Expressway section × Preventive maintenance treatment × Year.

To account for differences in maintenance technologies, the dataset was divided into three mutually exclusive subsets, namely crack filling (95 samples), surface sealing (75 samples), and overlay (69 samples). Separate predictive models were developed for each maintenance category. Accordingly, for modeling purposes, the crack filling dataset is used to develop and evaluate the XGBoost and FOA-XGBoost models, the surface sealing dataset is used to develop and evaluate the RF and HOA-RF models, and the overlay dataset is used to develop and evaluate the BPNN and PSO-BPNN models.

In the context of preventive maintenance decision-making for expressways, accurately predicting maintenance costs requires a systematic analysis of the interrelationship between pavement performance degradation mechanisms and actual engineering quantities. The development of a cost prediction model needs to incorporate factors such as pavement condition assessment metrics, maintenance workload, and environmental conditions. However, it is also necessary to consider the difficulty of collecting these data and the size of the dataset. Specifically, when the sample size is limited, incorporating an excessive number of input variables may lead to model overfitting, thereby diminishing its generalizability. Consequently, following a thorough evaluation of various influencing factors, five indicators were chosen to act as the model’s input features.

The foundation for the determinants influencing the cost indicators chosen in this study is outlined as follows. First, four principal sub-indicators from pavement condition assessment were incorporated. The PCI, as a composite indicator, encapsulates the overall structural integrity of the pavement; its deterioration is closely associated with increased consumption of crack-sealing materials. When the RDI surpasses a defined critical threshold, it often necessitates high-cost interventions such as milling and resurfacing. Variations in the RQI are indicative of smoothness deficiencies, which are associated with elevated equipment and labor costs for corrective surface treatments. Similarly, a diminished SRI typically triggers the application of anti-skid surface treatments, incurring additional process-related expenditures. Collectively, the integrated analysis of these four indicators provides a comprehensive characterization of the variations in material, equipment, and process selection associated with different types of pavement damage.

Second, the volume of maintenance work was included as a factor, given that the resource input per unit of maintenance is relatively stable. As a result, a near-linear relationship between maintenance workload and cost can be reasonably assumed. The definitions and representations of all variables are summarized in Table 2.

**Table 2:** Description of the dataset for the prediction model.

Variable type	Name	Description	Unit	Value Range
Input	PCI	Pavement Condition Index, reflecting overall pavement damage condition	—	0–100
	RQI	Road Quality Index, reflecting surface roughness derived from the International Roughness Index	—	0–100
	RDI	Rutting Depth Index, reflecting pavement deformation condition	—	0–100
	SRI	Skid Resistance Index, reflecting pavement friction performance	—	0–100
	Maintenance workload	Quantity of maintenance work performed for a given project	m/m²	Project-dependent
Output	Maintenance costs	Total cost of preventive maintenance for a project	CNY (Yuan)	Project-dependent

Before training the model, the raw sample data were subjected to standardized preprocessing procedures. Given the substantial differences in scale among pavement performance indicators (PCI, RQI, RDI, SRI), maintenance workload, and associated costs, all data were subjected to non-dimensionalization. Specifically, a logarithmic transformation was first applied, as shown in Eq (1). The transformed data were then normalized using the minimum-maximum method, as shown in Eq (2).


$$\begin{array}{c} \hat{x}=\ln(x+1) \end{array}$$ (1)

where *x* is the original value, $\hat{x}$ is the transformed value, and ln denotes the natural logarithm.


$$\begin{array}{c} \hat{z}=\frac{z-z_{\text{min}}}{z_{\text{max}}-z_{\text{min}}} \end{array}$$ (2)

where $\hat{z}$ is the normalised data; *z* is the original value of the feature indicator; *z*_min_ is the minimum value; *z*_max_ is the maximum value.

All preprocessing procedures were applied consistently across the dataset without additional filtering or artificial data augmentation. The complete raw dataset is provided in Supporting Information as S1 Table, and detailed descriptions of all variables are provided in S2 Table, where each record corresponds to the input and output variables used in the predictive models. Following data preprocessing, the dataset was randomly split into training and testing subsets, with 70% of the data used for training and 30% for testing. The predictive models for maintenance costs were developed using the training dataset, while the test dataset was used to evaluate model performance.

### 2.3. Model development

To enhance the accuracy and adaptability of the predictive models, this research strategically selected machine learning algorithms aligned with the specific characteristics of various preventive maintenance techniques, including crack filling, surface sealing, and overlay. The selection criteria incorporated considerations such as the technical maturity of each maintenance method, the volume of available historical data, and the rate of technological advancement within each field. Accordingly, three machine learning models were adopted: XGBoost, RF, and BPNN. Each model exhibits distinct advantages in terms of learning efficiency, interpretability, and suitability for handling complex engineering datasets. A summary of the justification for choosing each model is provided in Table 3.

**Table 3:** Justification for machine learning model selection.

Model	Applicable Maintenance Type	Maintenance Type Characteristics	Justification for Model Selection
XGBoost	Crack filling	Technically mature; large and stable dataset; slow technological change	Suitable for structured, high-frequency data associated with mature processes
RF	Surface sealing	Moderately mature; medium-sized dataset; rapid material updates	Well-suited for scenarios with medium data availability and changing material characteristics
BPNN	Overlay	Technologically evolving, limited, and less consistent dataset; dynamic development of techniques and materials	Appropriate for capturing complex cost dynamics in evolving engineering applications

#### 2.3.1. XGBoost.

XGBoost is an efficient machine learning algorithm constructed on the basis of gradient boosting decision trees (GBDT). Friedman [33] systematically described gradient boosting decision trees in 1999, laying the theoretical foundation for improving model performance through iterative residual fitting. Based on the GBDT theoretical framework, XGBoost has made breakthrough innovations and optimizations in several key technologies to improve model performance and computing speed [34,35]. The objective function design of XGBoost is based on the traditional gradient boosting framework and integrates regularization terms and second-order Taylor expansions, as shown in Eq (3).


$$\begin{array}{c} L(\phi )=\sum_{i=1}^{n}l(y_{i},{\hat{y}}_{i})+\sum_{k=1}^{K}\Omega (f_{k})=\sum_{j=1}^{T}\left[G_{j}w_{j}+\frac{1}{2}\left(H_{j}+\lambda \right)w_{j}^{2}\right]+\gamma T \end{array}$$ (3)

where L(ϕ) is the objective function; *n* stands for the quantity of training samples; $l\left(y_{i},{\hat{y}}_{i}\right)$ is the loss function, where *y*_*i*_ is the true label value of the *i* -th sample and ${\hat{y}}_{i}$ is its predicted value; $\Omega (f_{k})$ is the regularisation term for the tree, with *k* is the number of trees; *G*_*j*_ and *H*_*j*_ are the first-order and second-order derivative information related to the *j* -th leaf node, respectively; *w*_*j*_ is the weight of the leaf node; γ is the regularisation term of the leaf node; *T* is the number of leaf nodes.

#### 2.3.2. RF.

As a representative ensemble algorithm, RF takes decision trees as the base learners for its model construction. It is a typical application of the Bagging algorithm, which enhances the exactness and reliability of predictions through the construction and integration of multiple decision trees [36]. In this model, multiple training subsets are generated from the original dataset using bootstrap sampling. Each decision tree is constructed based on an independent bootstrap sample, which means that the training data for each tree differs, introducing diversity into the base learners. In comparison with a single decision tree, the random forest model significantly improves the model’s tolerance to noise and outliers while maintaining accuracy, thereby reducing the risk of overfitting [37,38]. The calculation formula is shown in Eq (4).


$$\begin{array}{c} \hat{y}=\frac{1}{K}\sum_{k=1}^{K}h_{k}(x) \end{array}$$ (4)

where *K* is the number of trees, *h*_*k*_(*x*) is the prediction result of the tree, and *x* is the input feature.

#### 2.3.3. BPNN.

BPNN is a multilayer feedforward neural network based on a feedforward network architecture. The backpropagation (BP) algorithm introduced by Rumelhart and McClelland in 1986, remains one of the most widely employed models in artificial neural network research [7]. The model is composed of input layer, hidden layer, and output layer. Input data is forwarded through multiple layers of neurons to generate prediction results. The error between the predicted value and the actual value is then calculated using the loss function, and the error is propagated backward from the output layer to the input layer via the chain rule, with the connection weights of the neurons updated layer by layer. The input layer of this study has five neurons, the hidden layer has eight neurons, and the output layer has a single neuron. Compared to traditional linear models, the capability to autonomously learn complex nonlinear mappings is possessed by BPNN. As long as the number of hidden layer neurons is sufficient, it can approximate any continuous function with arbitrary precision [39]. The main steps in learning and training a BPNN model are as follows:

1. Initialize the neural network, determine the number of nodes, weights, and thresholds for each layer, and select the Sigmoid function as the activation function
2. Calculate the output values whose definitions are shown in Eqs (5) and (6)
  $$\begin{array}{c} Y_{\ell }=\phi \left(\sum_{i=1}^{p}x_{i}\cdot \omega_{i\ell }-\beta_{\ell }\right) \end{array}$$ (5)
  $$\begin{array}{c} Z_{m}=\sum_{\ell =1}^{q}\left(Y_{\ell }\cdot \theta_{\ell m}\right)-\gamma_{m} \end{array}$$ (6)
  where $Y_{\ell }$ is the hidden layer output; *p* is the number of input layer nodes; $\omega_{i\ell }$ is the connection weight between the input layer and the hidden layer; $\beta_{\ell }$ is the bias from the input layer to the hidden layer. *Z*_*m*_ is the output layer output; $\theta_{\ell m}$ is the connection weight between the hidden layer and the output layer; $\gamma_{m}$ is the bias from the hidden layer to the output layer.
3. Calculate the error between the training set output value and the actual value.
4. Through error backpropagation training, the weights and thresholds of the input layer, hidden layer, and output layer in the neural network are continuously updated.
5. Check whether the error is less than the preset value. If it is, terminate the training and output the calculation results; if not, return to step 2.

### 2.4. Hybrid optimization algorithms and hyperparameter settings

**Fig 4:**
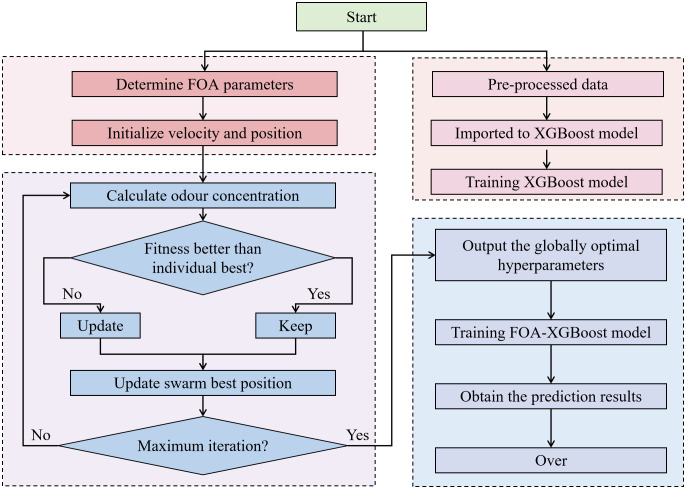
Prediction flow chart of FOA-XGBoost model.

**Fig 5:**
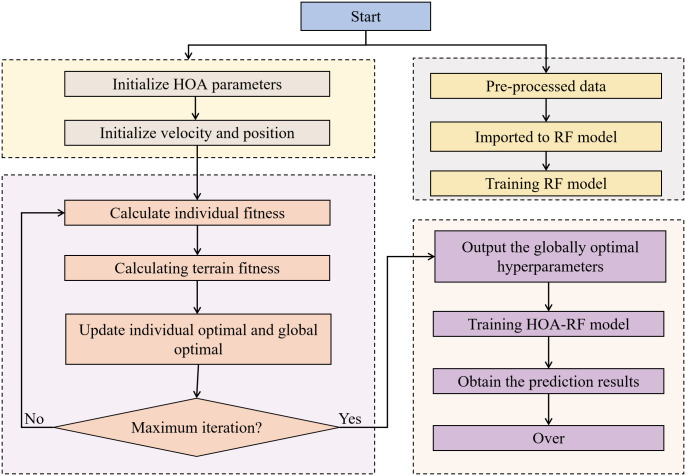
Prediction flow chart of the HOA-RF model.

**Fig 6:**
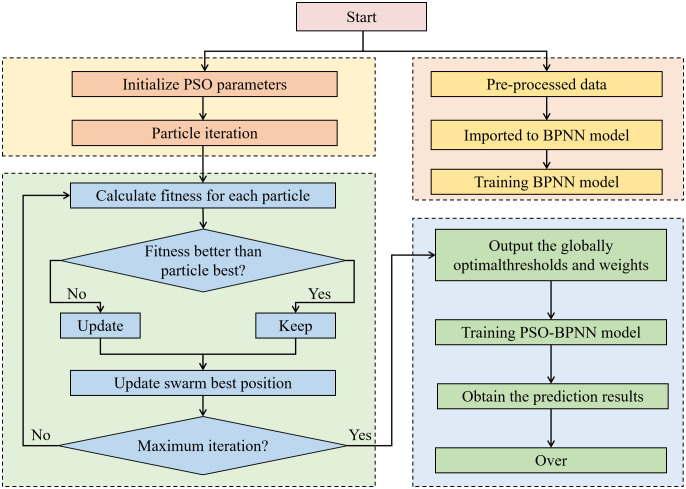
Prediction flow chart of the PSO-BPNN model.

Based on the dataset collected from 13 expressways in Xinjiang during the period from 2021 to 2025, predictive models were developed for three types of preventive maintenance measures: crack filling, surface sealing, and overlay. To improve model performance, meta-heuristic algorithms (FOA, HOA, and PSO) were employed for hyperparameter optimization, replacing conventional methods such as grid search. Three corresponding hybrid models were established, including FOA-XGBoost, HOA-RF, and PSO-BPNN. Their workflows are illustrated in Figs 4–6. In this framework, each candidate solution represents a set of hyperparameters of the base model, and the optimal configuration is obtained by minimizing a predefined objective function.

#### 2.4.1. FOA.

FOA is a meta-heuristic optimization algorithm based on olfactory-visual collaborative search, introduced by Pan, W. T. in 2012 [40]. The algorithm models the biological behavior of fruit flies by initially allowing each individual to explore its environment through random flight, each fruit fly relies on an optimized objective function to complete the position determination and optimization process.

For crack-filling maintenance measures, the FOA algorithm is used to optimize key hyperparameters of the XGBoost model, including the number of trees, maximum depth, learning rate, and subsample ratio. The initial population size of FOA is set to 20, and the maximum number of iterations is 100. The optimization process consists of the following steps:

Step 1: Initialization Phase

Randomly generate the initial positions of the fruit fly population:


$$\begin{array}{c} X_{i}=(x_{i1},x_{i2},\ldots ,x_{iD})\;(i=1,2,\ldots ,N) \end{array}$$ (7)

where *X*_*i*_ is the D-dimensional position vector of the *i*-th fruit fly, and *N* is the population size.

Step 2: Olfactory Search

Each fruit fly randomly flies based on its current position to generate a new position:


$$\begin{array}{c} {\hat{X}}_{i}=X_{i}+\text{Random}()+\text{StepSize} \end{array}$$ (8)

where *R* is a random vector with elements in the range [−1, 1], and *S* is the search step size.

Step 3: Calculate Smell Concentration

Evaluate the fitness by substituting the new position into the objective function:


$$\begin{array}{c} Smell_{i}=f(X_{i}) \end{array}$$ (9)

Each candidate hyperparameter set is used to train the XGBoost model, and the *R*^2^ is computed as the fitness value.

Step 4: Update Individual and Global Optima

If the current smell concentration is better than the historical record, update it as:


$$\begin{array}{c} P_{best,i}=X_{i}^{\prime }\;\text{if}\;Smell_{i}<Smell_{P_{best,i}} \end{array}$$ (10)

Compare the smell concentrations of all individuals and update the global best position:


$$\begin{array}{c} G_{best}=\text{arg}\min_{i=1}^{N}Smell_{P_{best,i}} \end{array}$$ (11)

Step 5: Visual Guidance

The fruit fly population converges toward the global best position to generate the next generation of positions:


$$\begin{array}{c} X_{i}(t+1)=G_{best}(t)+\text{Random}()\cdot \alpha \cdot \left|G_{best}(t)-X_{i}(t)\right| \end{array}$$ (12)

where α is the convergence coefficient, and the current iteration number is *t*.

The above steps are repeated until convergence or the maximum iteration number is reached.

#### 2.4.2 HOA.

HOA is a meta-heuristic algorithm designed to simulate the decision-making process of humans searching for the optimal path in complex terrain. The inspiration for this algorithm comes from the experience of humans hiking to the summit [41]. The algorithm uses terrain as the solution space, hikers choose their direction of movement and adjust their stride length based on their physical strength and terrain conditions. Then, hikers calculate their position based on the leading hiker and the group’s leading position, and generate new positions through local perturbations near the temporary position.

For surface sealing maintenance measures, HOA is adopted to optimize the hyperparameters of the RF model, including the number of trees, maximum depth, maximum features, and minimum samples split. The initial population size of HOA is set to 15, and the maximum number of iterations is 30. The optimization process consists of the following steps:

Step 1: Initialization Phase

Randomly generate the initial positions of the hikers to form the initial population $X=(x_{1},x_{2},\ldots ,x_{N})$.

Step 2: Energy Consumption and Recovery


$$\begin{array}{c} E_{i}(t)=E_{i}(t-1)\cdot \left(1-\beta \cdot \frac{f(x_{i}(t-1))-f_{min}(t-1)}{f_{max}(t-1)-f_{min}(t-1)+\epsilon }\right) \end{array}$$ (13)

$$\begin{array}{c} E_{i}(t)=E_{i}(t)+\gamma \cdot \left(1-\frac{f(x_{i}(t))-f_{min}(t)}{f_{max}(t)-f_{min}(t)+\epsilon }\right) \end{array}$$ (14)

*E*_*i*_(*t*) denotes the energy value of hiker *i* at the *t*-th iteration, β is *t*he energy consumption coefficient within the range (0,1), and γ is the energy recovery coefficient within the range (0,1). $f_{max}(t-1)$ and $f_{min}(t-1)$ represent the maximum and minimum fitness values at the (*t* − 1)-th iteration, respectively, and ϵ is a small positive constant to avoid numerical singularity.

Step 3: Movement Direction and Step Size Adjustment


$$\begin{array}{cc} v_{i}(t) & =\omega \cdot v_{i}(t-1)+c_{1}\cdot r_{1}\cdot \left(x_{ibest}(t-1)-x_{i}(t-1)\right)\\ & \;+c_{2}\cdot r_{2}\cdot \left(x_{gbest}(t-1)-x_{i}(t-1)\right) \end{array}$$ (15)

$$\begin{array}{cc} x_{i}(t) & =x_{i}(t-1)+v_{i}(t)\cdot \frac{E_{i}(t-1)}{\sum_{j=1}^{N}E_{j}(t-1)} \end{array}$$ (16)

*v*_*i*_(*t*) denotes the velocity vector, and *x*_*i*_(*t*) represents the updated position of hiker *i*. ω is the inertia weight, *c*_1_ and *c*_2_ are acceleration constants, while *r*_1_ and *r*_2_ are uniformly distributed random numbers within the range [0,1]. The term $\frac{E_{i}(t-1)}{\sum_{j=1}^{N}E_{j}(t-1)}$, defined as the energy proportion of hiker *i*, is used to dynamically adjust the step size during iteration.

Step 4: Terrain Fitness Calculation

Each hiker represents a candidate hyperparameter combination, and the fitness is evaluated based on the MSE of the RF model.

The position of each hiker is updated iteratively according to the exploration mechanism of HOA, and the optimal solution is obtained when the stopping criterion is satisfied.

#### 2.4.3 PSO.

PSO is a swarm intelligence optimization algorithm proposed by Kennedy and Eberhart in 1995 [42], based on the foraging behavior of flocks of birds in nature. This algorithm simulates the collaborative behavior of flocks of birds, treating each solution in the search space as a particle. Each particle has its own position, velocity, and fitness value. The update of its velocity not only combines its own experience but also considers the information exchange among the group.

For overlay maintenance measures, PSO is used to optimize the hyperparameters of the BPNN model, including the number of hidden neurons, learning rate, and training iterations.The initial particle population is set to 20, and the maximum number of iterations is 30. The inertia weight is set to 0.9. The optimization process is defined as follows:

Step 1: Initialization Phase

Randomly generate the position and velocity of each particle:


$$\begin{array}{c} X_{i}=(x_{i1},x_{i2},\ldots ,x_{iD}) \end{array}$$ (17)

$$\begin{array}{c} V_{i}=(v_{i1},v_{i2},\ldots ,v_{iD})\;(i=1,2,\ldots ,N) \end{array}$$ (18)

Where *X*_*i*_ denotes the *d*-dimensional position of the *i*-th particle, $V_{i}$ represents the *d*-dimensional flight velocity of the *i*-th particle, and *N* is the population size.

Step 2: Velocity and Position update


$$\begin{array}{cc} v_{ij}(t+1) & =w(t)v_{ij}(t)+c_{1}\cdot r_{1}\cdot \left(P_{ibest}(t)-x_{ij}(t)\right)\\ & \;+c_{2}\cdot r_{2}\cdot \left(G_{best}(t)-x_{ij}(t)\right) \end{array}$$ (19)

$$\begin{array}{cc} x_{ij}(t+1) & =x_{ij}(t)+v_{ij}(t+1) \end{array}$$ (20)

Where *c*_1_ and *c*_2_ are acceleration constants, *r*_1_ and *r*_2_ are uniformly distributed random numbers within the range [0,1], and *v*_*ij*_ is the velocity of the particle, with $v_{ij}\in [-v_{\max},v_{\max}]$, where $v_{\max}$ is a constant. Additionally, *x*_*ij*_ is the position of the particle, with $x_{ij}\in [-x_{\max},x_{\max}]$, where $x_{\max}$ is a constant.

Step 3: Fitness evaluation and update of optimal solutions

The iterative process continues until the convergence condition is met.

#### 2.4.4. Hyperparameter settings.

The search ranges and initial configurations of the hyperparameters for all models are summarized in Table 4. These ranges were determined based on prior studies and empirical experience to ensure sufficient coverage of the parameter space. During the optimization process, all hyperparameters were searched within these predefined ranges.

**Table 4:** ML model hyperparameter selection.

Model	Hyperparameters	Default value	Optimization algorithm	Set range
XGBoost	Number of trees	100	FOA	50-500
	Max depth	4		2 - 8
	Rate of learning	0.05		0.01 - 0.1
	Subsample	0.8		0.5 - 1
RF	Number of trees	1000	HOA	50 - 200
	Max depth	8		3 - 20
	Max features	0.5		0.1 - 0.9
	Min samples split	2		2 - 10
BPNN	The activation function of the hidden layer	Sigmoid	PSO	/
	Hidden size	8		2 - 20
	Rate of learning	0.1		0.0001 - 0.5
	The number of iterations	1000		100 - 2000

### 2.5. Model evaluation

To objectively assess model performance, this study employs multiple quantitative metrics to measure prediction accuracy, while a nested cross-validation scheme is utilized to ensure reliable and robust model validation.

#### 2.5.1. Evaluation metrics.

Model evaluation serves as an effective approach to facilitate the understanding of model performance and enhance the interpretability of model results. As this study focuses on a regression-based prediction task, with the objective of comparing the generalization performance of different models in preventive maintenance cost prediction, five evaluation metrics were selected, including R², MAE, MSE, RMSE, and MAPE. This selection avoids the one-sidedness of relying on a single metric and provides a more comprehensive perspective for the interpretation of model performance.

From the perspective of variance explanation, R² quantifies the extent to which the model accounts for variability in the data. MAE is robust to outliers and insensitive to extreme values, and can intuitively reflect the average level of prediction error. MAPE removes the effects of differing units by expressing errors as relative percentages. MSE works by calculating the average of squared differences between predicted and actual values and serves as a metric for measuring overall error magnitude. RMSE incorporates squared errors and imposes greater penalties on larger deviations, thereby reflecting the model’s performance with respect to extreme observations. These indicators are shown in Eqs (21)-(25)


$$\begin{array}{c} R^{2}=1-\frac{\sum_{i=1}^{n}{\left(y_{i}-{\hat{y}}_{i}\right)}^{2}}{\sum_{i=1}^{n}{\left(y_{i}-\bar{y}\right)}^{2}} \end{array}$$ (21)

$$\begin{array}{c} MAE=\frac{\sum_{i=1}^{n}\left|y_{i}-{\hat{y}}_{i}\right|}{n} \end{array}$$ (22)

$$\begin{array}{c} MAPE=\frac{1}{n}\sum_{i=1}^{n}\left|\frac{y_{i}-{\hat{y}}_{i}}{y_{i}}\right|\times 100\% \end{array}$$ (23)

$$\begin{array}{c} MSE=\frac{1}{n}\sum_{i=1}^{n}{\left(y_{i}-{\hat{y}}_{i}\right)}^{2} \end{array}$$ (24)

$$\begin{array}{c} RMSE=\sqrt{\frac{1}{n}\sum_{i=1}^{n}{\left(y_{i}-{\hat{y}}_{i}\right)}^{2}} \end{array}$$ (25)

#### 2.5.2. Nested cross-validation.

Nested cross-validation is a statistical method for evaluating the performance of machine learning models. Compared with a single data split, it uses repeated data partitioning to assess model performance on different subsets, thereby reducing the influence of sample randomness on the evaluation results. Considering the limited sample size and the further reduction in data volume after grouping by different maintenance measures, a nested cross-validation strategy was adopted to evaluate the reliability of model evaluation.

In this study, a 10-fold outer cross-validation and 3-fold inner cross-validation framework was employed. Specifically, the dataset was first divided into ten mutually exclusive folds. In each outer iteration, one fold was used as the test set and the remaining nine folds were used as the outer training set. The outer test fold was used exclusively for final performance evaluation and was not involved in parameter optimization.

Within each outer training set, hyperparameter optimization was performed using the corresponding metaheuristic algorithms (FOA, HOA, and PSO). During this process, a 3-fold inner cross-validation procedure was used to evaluate candidate hyperparameter solutions. Specifically, the outer training data were further divided into three folds, and the average performance across the three inner folds was used as the fitness value for the metaheuristic optimization process.

After the optimal hyperparameter combination was identified within the inner loop, the model was retrained on the outer training data and then evaluated on the corresponding outer test fold using the predefined evaluation metrics. This process was repeated across all ten outer folds, and the final model performance was obtained by averaging the results over the outer folds.

Based on the cross-validation results, the confidence intervals (CI) were estimated using a bootstrap resampling approach, which does not rely on distributional assumptions. The mean ± standard deviation and 95% confidence intervals were then calculated for each performance metric to quantify the variability and prediction uncertainty of the models.

### 2.6. Statistical significance testing

To evaluate whether the performance differences between the optimized hybrid models and their baseline counterparts are statistically significant, the Wilcoxon signed-rank test was conducted based on cross-validation results, where key performance metrics (e.g., MAE and RMSE) obtained from each fold were treated as paired samples for model comparison. Statistical significance was determined at a predefined level of α=0.05 [43].

In addition, effect size was calculated using the rank-biserial correlation to quantify the magnitude of performance differences between models, with its absolute value ranging from 0 to 1, where larger values indicate a stronger difference between paired observations.

### 2.7. Influencing factor analysis and sensitivity analysis

To clarify the driving mechanism of input parameters on the prediction results of highway preventive maintenance cost, and further verify the robustness and interpretability of the proposed prediction model, two complementary analyses were conducted in this study: feature importance evaluation and sensitivity analysis. The former quantified the relative contribution of each input feature to the maintenance cost prediction output, while the latter examined the response law of model predicted values to systematic variations in a single input variable.

#### 2.7.1. Feature importance evaluation.

Feature importance evaluation is a critical component in the development of predictive models using machine learning. The contribution of each input variable to the target outcome is quantified, and this enables the model to focus on the features that are most relevant. This process reduces the risk of overfitting, enhances predictive performance and generalization capability, and contributes to improved model interpretability and accuracy.

The core purpose of feature importance analysis is to identify features that have significant predictive power in driving the target variable. Quantifying the contribution of each input feature to the prediction outcome helps us gain a deeper understanding of how different features influence the prediction results, thereby enhancing the model’s interpretability and making the model more straightforward to comprehend. On the other hand, by emphasizing the most critical input features to improve the model’s performance. This approach effectively reduces the risk of overfitting and enhances the model’s capacity to generalize to novel data. In addition, by retaining only the most relevant variables, this process streamlines the model architecture and reduces computational complexity. The following are some commonly used feature importance evaluation methods:

1. Model-intrinsic Explanation Methods
  - Tree-based Models: Feature importance is determined by such ensemble methods as Random Forests, Gradient Boosting Machines, and XGBoost, where the contribution of each feature to the optimization objective is quantified in the process of node splitting.
  - Linear Models: The importance of features in linear models like linear regression and logistic regression is estimated by examining feature coefficients’ absolute values, which denote the marginal contribution of each variable to the model output.
2. Model-agnostic Explanation Methods
  - Permutation Importance: This method works by randomly shuffling the values of individual features, followed by re-evaluating the model’s performance on the shuffled dataset using the same model. The degree of decline in model performance measures the importance of each feature.
  - SHAP (Shapley Additive exPlanations) values: SHAP is a method for gaining insight into specific instances by measuring the contribution of each feature, proposed by Lundberg and Lee in 2017 [44]. Grounded in cooperative game theory, SHAP calculates the fair contribution value of each feature to the prediction, which serves as a measure of feature importance.

#### 2.7.2. Sensitivity analysis.

In this study, a sensitivity analysis was conducted to evaluate the influence of input variables on model predictions. A one-at-a-time (OAT) approach was adopted, in which each input variable was individually perturbed while keeping all other variables constant. Specifically, each input variable was varied by ±5%, ± 10%, ± 15%, and ±20% relative to its baseline value. The selected perturbation range reflects small to moderate variations in the input variables.

For each perturbation level, the pre-trained optimal model was used to generate new predictions of maintenance cost, and the corresponding change in the predicted output (y) was recorded. The sensitivity of the model to each input variable was then evaluated according to the magnitude of the relative output variation.

## 3. Case study

### 3.1. Data description

The dataset used in this study is derived from actual maintenance data of Xinjiang expressway pavements, covering three types of preventive maintenance projects: crack filling, surface sealing, and overlay. It includes a total of 239 valid samples, with 95 samples for crack filling, 75 for surface sealing, and 69 for overlay. The core feature variables of the dataset include pavement performance indicators PCI, RQI, RDI, and SRI, all of which comply with the JTG 5210–2018 “Highway Technical Condition Evaluation Standard.” Additionally, the dataset contains maintenance quantities, with maintenance cost as the target variable. All data were log-transformed and normalized using Eqs (1) and (2) to eliminate dimensional effects and enhance model training stability.

### 3.2. Comparative analysis of the results of the prediction models

The experiment was run on a computer with an AMD Ryzen (TM) 5 5500U CPU @ 2.1 GHz, 64-bit OS, and 64× architecture. To ensure complete experimental reproducibility, a fixed random seed of 42 was set for all Python scripts, model training, and algorithm optimization processes. This study first established three machine learning models (XGBoost, RF, and BPNN) using Python software to predict preventive maintenance costs for expressways. Then, meta-heuristic algorithms compatible with the models were introduced to optimize the model hyperparameters, resulting in hybrid models (FOA-XGBoost, HOA-RF, and PSO-BPNN). This approach, which combines intelligent optimization algorithms with machine learning models, demonstrated superior predictive accuracy compared to the individual models. The summary of the evaluation metrics for the predictive performance of all six models is shown in Table 5.

**Table 5:** Six methods for evaluating metrics on datasets.

Ensemble Model	*R* ^2^	MAE	MSE	RMSE	MAPE (%)
XGBoost	0.8096	0.0642	0.0076	0.0873	4.85
**FOA-XGBoost**	**0.8622**	**0.0552**	**0.0055**	**0.0742**	**4.13**
RF	0.8443	0.0557	0.0046	0.0679	3.32
**HOA-RF**	**0.8921**	**0.0457**	**0.0032**	**0.0565**	**2.78**
BPNN	0.9137	0.0562	0.0053	0.0730	3.59
**PSO-BPNN**	**0.9277**	**0.0419**	**0.0045**	**0.0668**	**2.64**

Overall, in crack-filling maintenance measures, the evaluation metrics for the XGBoost model were R² = 0.8096, MAE = 0.0642, MSE = 0.0076, RMSE = 0.0873, and MAPE = 4.85%. After optimization using the FOA algorithm, the performance of the FOA-XGBoost prediction model improved significantly, R² = 0.8622, MAE = 0.0552, MSE = 0.0055, RMSE = 0.0742, MAPE = 4.13%. The results indicate that FOA plays a positive role in improving model generalization ability and error control. Among the surface sealing maintenance measures, the RF model demonstrated superior stability, with R² = 0.8443, MAE = 0.0557, MSE = 0.0046, RMSE = 0.0679, and MAPE = 3.32%. After HOA optimization, the model performance was further enhanced. The HOA-RF model achieved R² = 0.8921, MAE = 0.0457, MSE = 0.0032, RMSE = 0.0565, and MAPE = 2.78%. These results substantiate the efficacy of the HOA algorithm in parameter tuning for tree models. Among overlay maintenance measures, the BPNN model demonstrated optimal performance under the optimization of the PSO algorithm. The PSO-BPNN model achieved R² = 0.9277, MAE = 0.0419, MSE = 0.0045, RMSE = 0.0668, and MAPE = 2.64%. Compared to the unoptimized BPNN (R² = 0.9137, MAE = 0.0562, MSE = 0.0053, RMSE = 0.0730, MAPE = 3.59%), the PSO algorithm significantly enhances the model’s nonlinear fitting capability by optimizing the network structure parameters.

The findings demonstrate that, compared with traditional machine learning models, the MAE, MSE, RMSE, and MAPE of the prediction model integrating meta-heuristic algorithms are all reduced, while R² is improved, which underscores the superior predictive performance of the hybrid model.

**Fig 7:**
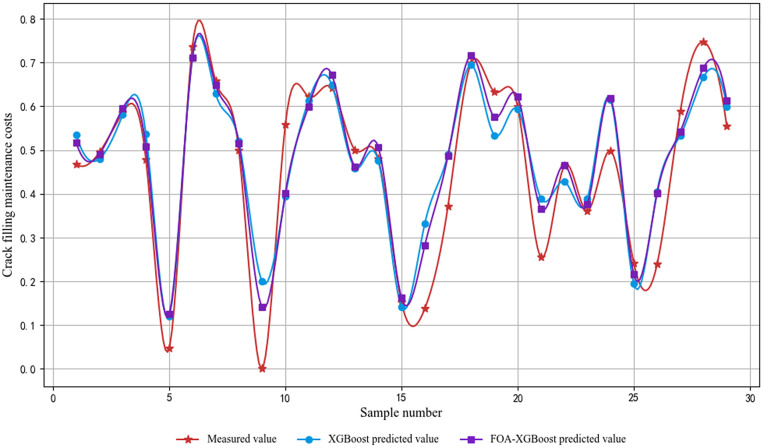
Comparison of predicted and actual costs for crack-filling maintenance measure.

**Fig 8:**
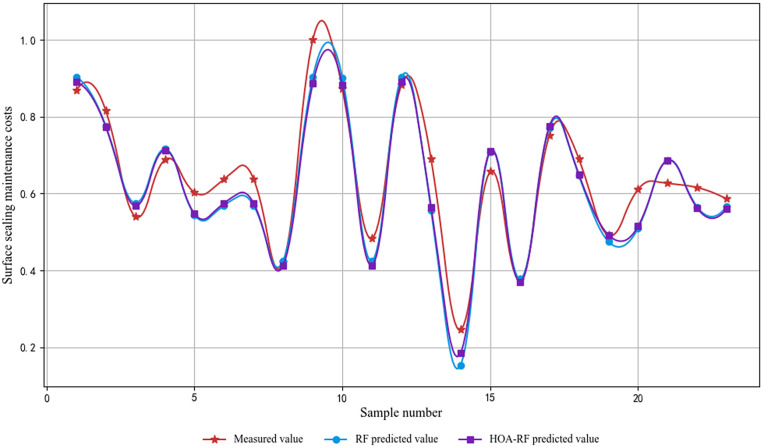
Comparison of predicted and actual costs for surface sealing maintenance measure.

**Fig 9:**
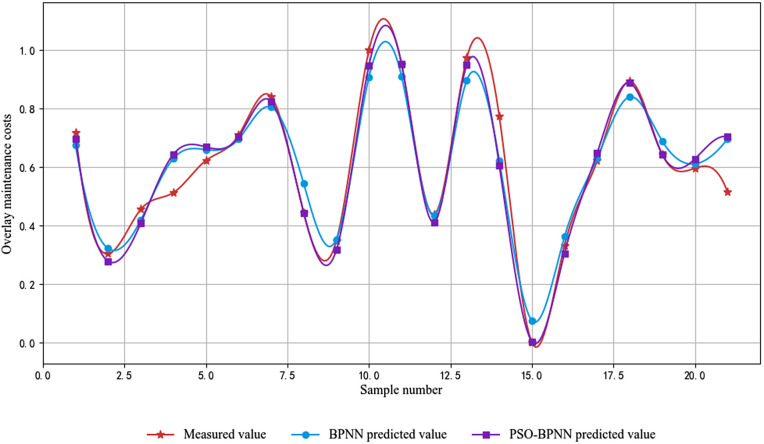
Comparison of predicted and actual costs for the overlay maintenance measure.

**Fig 10:**
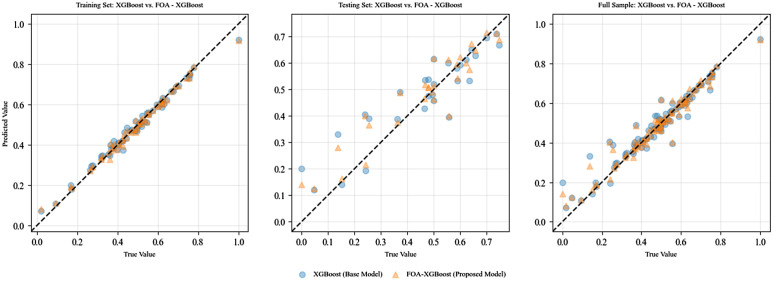
Crack filling maintenance costs prediction: XGBoost vs. FOA-XGBoost across data splits.

**Fig 11:**
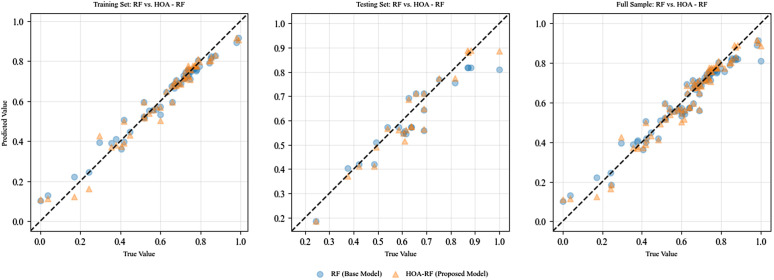
Surface sealing maintenance costs prediction: RF vs. HOA-RF across data splits.

**Fig 12:**
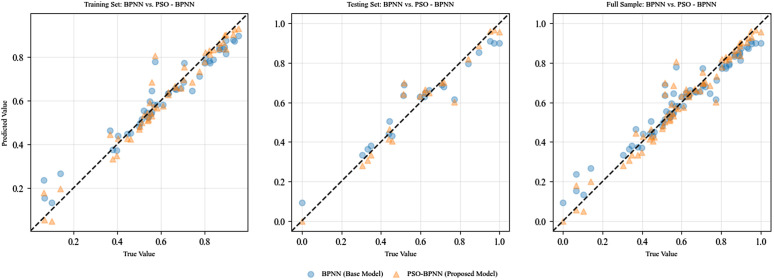
Overlay maintenance costs prediction: BPNN vs. PSO-BPNN across data splits.

Figs 7–9 show the prediction results obtained using the XGBoost, FOA-XGBoost, RF, HOA-RF, BPNN, and PSO-BPNN models. Through the comparison of predicted values and actual values, a cost prediction comparison chart was created for the three maintenance measures. Figs 10–12 show the scatter plots of the prediction models for the three maintenance measures. The predictive performance of the FOA-XGBoost, HOA-RF, and PSO-BPNN models shows measurable improvements relative to the original base models (XGBoost, RF, and BPNN).

### 3.3. Wilcoxon signed-rank test results of predictive models

The statistical significance of performance differences between each optimized model and its baseline counterpart was assessed via the Wilcoxon signed-rank test, using results from 10-fold cross-validation. The full test results, including effect sizes and error reduction rates, are presented in Table 6.

**Table 6:** Statistical comparison results between baseline and optimized models.

Model Comparison	Metric	*W*	p-value	*r*	Error Reduction(%)
XGBoost vs FOA − XGBoost	MAE	16	0.275	0.418	7.4
	MSE	17	0.322	0.382	8.4
	RMSE	16	0.275	0.418	4.5
	MAPE	15	0.232	0.455	6.4
RF vs HOA − RF	MAE	17	0.322	0.382	3.7
	MSE	22	0.625	0.200	−2.2
	RMSE	24	0.770	0.127	0
	MAPE	18	0.375	0.345	2.0
BPNN vs PSO−BPNN	MAE	8	0.049	0.709	33.3
	MSE	8	0.049	0.709	42.9
	RMSE	7	0.037	0.745	29.3
	MAPE	8	0.049	0.709	34.5

In the crack filling treatment subset, the FOA-XGBoost model achieved consistent performance gains over the baseline XGBoost across all four error metrics, with relative error reductions between 4.5% and 8.4%. While these improvements did not meet the pre-defined threshold for statistical significance (*p* > 0.05), the moderate rank-biserial correlation effect sizes (r = 0.382–0.455) demonstrate a non-trivial, stable improvement trend across all cross-validation folds. This finding suggests that FOA optimization provides tangible benefits to predictive performance for crack filling modeling.

For the surface sealing treatment subset, the HOA-RF model delivered marginal refinements in mean error metrics, with a 3.7% reduction in MAE and 2.0% reduction in MAPE, while matching the baseline RF model’s performance in squared error metrics (MSE, RMSE). No statistically significant differences were detected between the two models (*p* > 0.05), and the effect sizes ranged from 0.127 to 0.382, corresponding to small-to-moderate practical effects. This performance profile indicates that HOA optimization primarily reduces average prediction deviations, while maintaining robustness to extreme outliers.

In the overlay treatment subset, the PSO-BPNN model demonstrates substantial improvements over the baseline BPNN model across all evaluation metrics, with error reductions ranging from 29.3% to 42.9%. These improvements are statistically significant (*p* < 0.05) and are accompanied by large effect sizes (r = 0.709–0.745), indicating a strong and consistent enhancement in predictive performance. This highlights the effectiveness of PSO optimization in improving both accuracy and robustness for overlay-related predictions.

Overall, all three meta-heuristic optimization strategies exhibited varying degrees of performance benefits within their corresponding treatment-specific subsets, with noticeable differences in improvement magnitude and statistical robustness. Specifically, FOA optimization achieved stable and consistent performance gains for crack filling prediction modeling; HOA optimization yielded marginal improvements in mean absolute and percentage error metrics, while maintaining comparable performance to the baseline RF model in squared error metrics for surface sealing applications; and PSO optimization produced the most substantial and statistically significant improvements across all metrics for overlay treatment modeling.

### 3.4. Cross-validation experimental results

To further enhance the reliability of model performance evaluation and mitigate potential overfitting risks, this study follows the 10-fold nested cross-validation framework detailed in Section 2.5.2 to validate all baseline and optimized models for different maintenance measures. The predictive performance of different models under three maintenance measures is summarized in Table 7. The results are reported as the mean ± standard deviation (SD), with the corresponding 95% confidence intervals (CI) provided in parentheses. To ensure full transparency and reproducibility, the fold-wise performance metrics for all six models across the 10-fold nested cross-validation are provided in S3 Table.

**Table 7:** Performance comparison of predictive models across three maintenance measures.

Maintenance measures	Model	MAE	MAPE (%)	MSE	RMSE
Crack Filling	XGBoost	0.068 ± 0.010 (0.056–0.083)	4.819 ± 0.811 (3.985–6.169)	0.008 ± 0.002 (0.005–0.011)	0.088 ± 0.014 (0.071–0.105)
	FOA-XGBoost	0.063 ± 0.011 (0.045–0.077)	4.509 ± 0.941 (3.405–6.373)	0.007 ± 0.003 (0.004–0.011)	0.084 ± 0.016 (0.063–0.105)
Surface Sealing	RF	0.054 ± 0.019 (0.022–0.093)	3.511 ± 1.311 (1.354–5.990)	0.005 ± 0.003 (0.001–0.012)	0.068 ± 0.022 (0.025–0.109)
	HOA-RF	0.052 ± 0.018 (0.025–0.091)	3.441 ± 1.222 (1.542–5.854)	0.005 ± 0.003 (0.001–0.012)	0.068 ± 0.022 (0.028–0.109)
Overlay	BPNN	0.063 ± 0.021 (0.036–0.104)	4.208 ± 1.569 (2.068–7.228)	0.007 ± 0.004 (0.002–0.015)	0.082 ± 0.026 (0.040–0.121)
	PSO-BPNN	0.042 ± 0.019 (0.020–0.075)	3.115 ± 1.328 (1.150–5.439)	0.004 ± 0.004 (0.001–0.012)	0.058 ± 0.028 (0.023–0.108)

The experimental results show that the three optimized models (FOA-XGBoost, HOA-RF, and PSO-BPNN) achieved varying degrees of performance benefits relative to their corresponding baseline models in their respective maintenance scenarios. Specifically, the PSO-BPNN model achieved lower mean errors and narrower confidence intervals across all evaluation metrics, while the FOA-XGBoost and HOA-RF models maintained comparable stability to their baseline models while delivering partial improvements in prediction accuracy.

For the crack filling scenario, the FOA-XGBoost model achieved consistent reductions in all four error metrics compared with the standard XGBoost model, with the mean MAE decreasing from 0.068 to 0.063 and mean MAPE decreasing from 4.819% to 4.509%, while the SD and 95% CI ranges remained comparable to the baseline model. For the surface sealing scenario, the HOA-RF model delivered marginal reductions in mean MAE (from 0.054 to 0.052) and MAPE (from 3.511% to 3.441%), with the mean MSE and RMSE remaining consistent with the conventional RF model. For the overlay maintenance scenario, the PSO-BPNN model achieved the best overall performance among all tested models, with the lowest mean values of MAE (0.042 ± 0.019), MAPE (3.115 ± 1.328%), MSE (0.004 ± 0.004), and RMSE (0.058 ± 0.028), alongside narrower confidence intervals than the baseline BPNN model.

The analysis of the above results indicates that the proposed metaheuristic-optimized models deliver scenario-specific performance improvements within the scope of this study. Meanwhile, the MAPE for each maintenance measure is maintained below 5%, which demonstrates that the three models achieve prediction accuracy and stable generalization behavior. Such consistent performance across cross-validation folds further supports the feasibility of the proposed modeling framework for maintenance cost prediction in this study.

### 3.5. Results of influencing factor and sensitivity analyses

**Fig 13:**
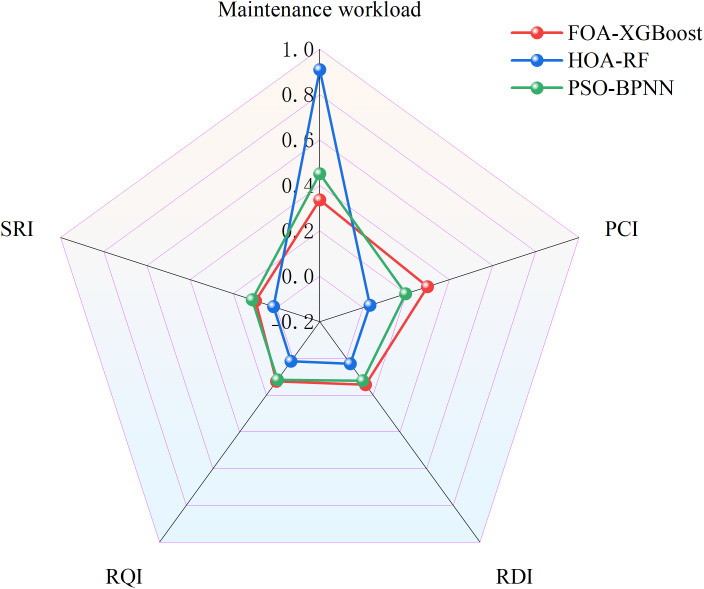
The relative importance of the input variables in three models.

**Fig 14:**
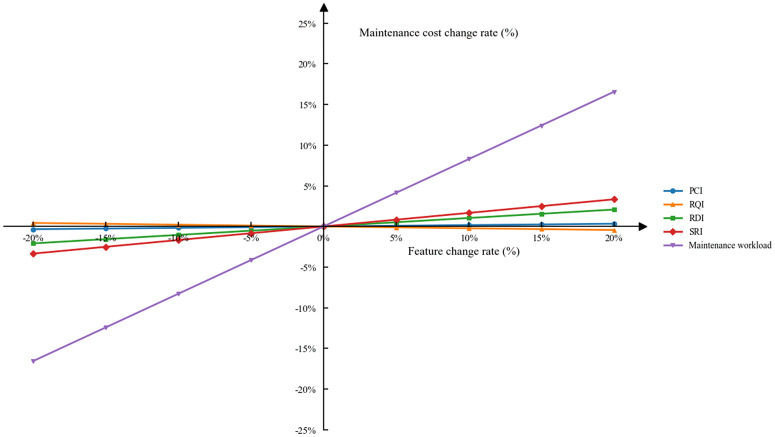
The sensitivity analysis results of the FOA-XGBoost model for maintenance cost prediction.

**Fig 15:**
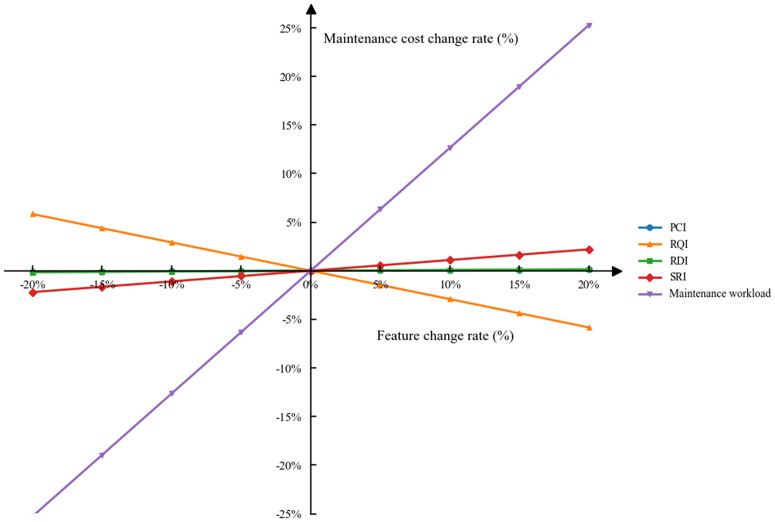
The sensitivity analysis results of the HOA-RF model for maintenance cost prediction.

**Fig 16:**
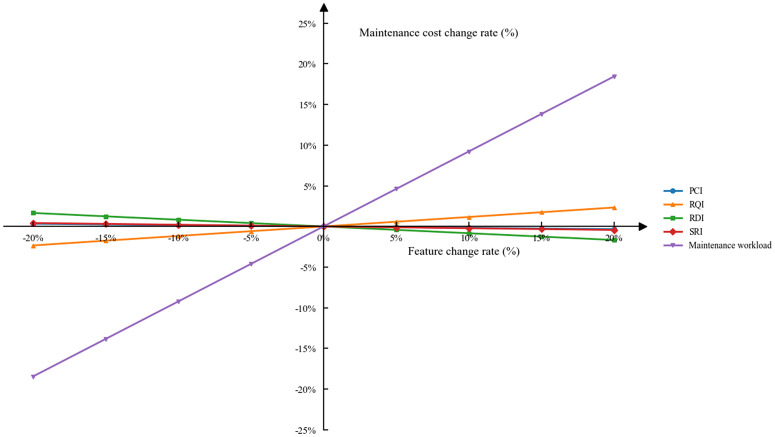
The sensitivity analysis results of the PSO-BPNN model for maintenance cost prediction.

Feature importance analysis was conducted to quantify the contribution of each predictor to preventive maintenance cost and to identify the key influencing factors of the proposed models. To further examine the reliability of the feature importance results and the marginal response of predicted cost to input variations, a single-factor sensitivity analysis based on the OAT approach was performed. Because separate predictive models were developed for crack filling, surface sealing, and overlay measures, the inherent feature importance functions of the XGBoost and RF models were used for crack filling and surface sealing, while permutation importance was adopted for the BPNN model in the overlay scenario. The corresponding feature importance rankings are shown in Fig 13, and the sensitivity response curves of the FOA-XGBoost, HOA-RF, and PSO-BPNN models are presented in Figs 14–16.

The combined results suggest that maintenance workload is the most influential factor affecting preventive maintenance costs across all three maintenance measures. This conclusion is also supported by the sensitivity analysis, in which maintenance workload consistently exhibits the largest response magnitude among all input variables. Specifically, a ± 20% variation in maintenance workload leads to an approximate ±16.5% change in predicted cost for crack filling, ± 25% for surface sealing, and ±18% for overlay. These results indicate that maintenance workload is a major driver of cost variation and should be considered in maintenance planning and budget allocation.

For different maintenance measures, the influencing factors of preventive maintenance costs exhibit distinct patterns. For crack-filling measures, PCI shows a relatively important contribution and a clear positive response to predicted cost. For surface sealing measures, the overall contribution of pavement performance indicators is relatively limited, whereas RQI shows the most notable effect among condition-related variables. For overlay measures, RDI, RQI, and SRI all contribute to cost variation to different extents, with RDI showing a negative response and RQI showing a positive response. These findings are consistent with the engineering characteristics of different maintenance treatments, in which construction complexity, structural condition, ride quality, and skid-related performance jointly influence maintenance demand and cost.

Overall, the integration of feature importance and sensitivity analysis provides a more comprehensive understanding of the driving mechanisms underlying preventive maintenance costs for expressways. On the one hand, it captures the global contribution of each input feature to cost prediction; on the other hand, it quantifies the local response of predicted cost to variations in individual factors. This dual perspective enhances the interpretability of the predictive models and offers targeted, quantitative guidance for cost control, maintenance strategy optimization, and efficient allocation of maintenance resources across different types of preventive maintenance measures.

## 4. Conclusion

Firstly, this study collected experimental data on key pavement performance indicators, including pavement damage, smoothness, rutting, skid resistance, and maintenance workload volume from a five-year preventive maintenance project for expressways in Xinjiang. Secondly, predictive models were developed for different maintenance interventions. For crack filling maintenance measures, the FOA-XGBoost model was constructed and contrasted with the XGBoost model. For surface sealing maintenance measures, the HOA-RF model was constructed and contrasted with the RF model. For overlay maintenance measures, the PSO-BPNN model was constructed and contrasted with the BPNN model. Following model development, a sequence of vital analyses was conducted on the input features that affect changes in preventive maintenance costs. Finally, cross-validation experiments served to assess the performance and robustness of the three hybrid models.

The detailed research findings are presented as follows: (1) Six independent models were employed to forecast preventive maintenance costs. The results from the experiment showed that the FOA-XGBoost model for crack filling maintenance measures had better predictive performance than the XGBoost model, with evaluation metrics of R² = 0.8622, MAE = 0.0552, MSE = 0.0055, RMSE = 0.0742, and MAPE = 4.13%. The HOA-RF model for surface sealing maintenance measures outperformed the RF model, with evaluation metrics of R² = 0.8921, MAE = 0.0457, MSE = 0.0032, RMSE = 0.0565, and MAPE = 2.78%. The predictive performance of the PSO-BPNN model for overlay maintenance measures is superior to that of the BPNN model, with evaluation metrics of R² = 0.9277, MAE = 0.0419, MSE = 0.0045, RMSE = 0.0668, and MAPE = 2.64%. (2) Under the nested cross-validation framework, the metaheuristic-optimized models showed consistent performance improvements compared to their corresponding baseline models across different maintenance scenarios. In particular, reductions in prediction errors and relatively stable performance were observed across validation folds, indicating that the proposed models maintain reliable predictive capability under the investigated conditions. (3)In addition, statistical analysis based on the Wilcoxon signed-rank test was conducted to further examine the performance differences between the optimized and baseline models. Meanwhile, feature importance and sensitivity analyses provide additional insights into the factors influencing maintenance costs. These analyses indicate that maintenance workload is an important influencing factor, while different maintenance measures exhibit varying sensitivities to pavement performance indicators. Specifically, its contribution rates to crack filling, surface sealing, and overlay maintenance measures were 33.61%, 90.96%, and 45.15%, respectively. Together, these findings contribute to a more comprehensive understanding of the proposed models within the scope of this study.

However, this study has certain limitations: (1) This study concentrates on preventive maintenance projects for expressways in Xinjiang, where the systematic collection of maintenance data has been conducted over a relatively brief period, making it challenging to construct a complete and continuous time-series dataset. Therefore, time-series data were excluded from the analysis. (2) The proposed model can only explain some of the characteristics that influence the target variable, and there are also external factors, such as maintenance material prices and regional construction costs, that affect the outcomes. (3) The data in this study are primarily concentrated in a specific region. Although the proposed model demonstrates high accuracy and stability within this context, its applicability to other settings may be limited, thereby constraining its generalizability.

With the aim of improving the model’s generalizability and accuracy, future research should address the following areas:

1. Further integration of expressway maintenance data from regions with more diverse climatic conditions and socioeconomic backgrounds, such as southern China and other countries worldwide, would enable more robust validation and refinement of the hybrid model proposed in this study, thereby enhancing its generalizability and practical utility for infrastructure management in broader contexts. Simultaneously, collecting long-term monitoring data and incorporating macroeconomic variables, together with time-series models such as LSTM or ARIMA-based hybrid approaches, to enable dynamic maintenance cost prediction over longer life cycles.
2. With the expansion of maintenance datasets, model performance and computational efficiency can be improved by innovating existing algorithms or developing new ones. In large datasets, incorporating deep learning for feature extraction can further enhance model performance.
3. The inclusion of environmental factors and regional policy variables is recommended, as they significantly impact maintenance cost variations and can inform tailored maintenance plans and resource allocation strategies for different regions.
4. Converting cost data from different years to the same base period using price indices ensures that cost estimates in maintenance plans are closer to reality. This adjustment allows the model’s interpretation of maintenance cost variability to transition from a static to a dynamic framework, providing a more scientific quantitative basis for managing maintenance costs throughout the entire lifecycle.

In practical applications, additional considerations are also necessary. When applied to public resource allocation, issues related to transparency, interpretability, and decision accountability should be carefully addressed. The integration of explainable artificial intelligence (XAI) techniques can help clarify the contribution of input features and improve model interpretability. At the same time, the proposed models are intended to serve as decision-support tools rather than fully automated decision-making systems, and human oversight remains essential to ensure responsible and accountable use of maintenance resources.

From an application perspective, the proposed hybrid models can be integrated into existing Pavement Management Systems (PMS) as a cost prediction module. Once maintenance needs are identified within the PMS, the appropriate model can be selected to generate cost estimates based on key pavement performance indicators. This data-driven approach can support preliminary budget planning, facilitate a transition from reactive to preventive maintenance strategies, and improve cost-effectiveness over the entire lifecycle. Furthermore, such integration provides a pathway for embedding data-driven cost estimation into existing decision-support workflows within infrastructure management agencies.

In summary, this study contributes to improving the prediction of preventive maintenance costs for expressways. By addressing the limitations of this study and exploring the suggested future research directions, the prediction model’s accuracy and universality can be enhanced.

## Supporting information

S1 Table Raw dataset of preventive maintenance projects. The raw data of pavement technical condition indicators, maintenance workload, and costs for multiple expressway sections with preventive maintenance.
(DOCX)

S2 Table Metadata and descriptive statistics of model variables. The metadata and descriptive information of all variables included in the raw dataset and predictive models, including definitions, units, value ranges.
(DOCX)

S3 Table Nested cross-validation results for predictive models. The 10-fold nested cross-validation outcomes for six predictive models, showing fold-specific performance metrics.
(DOCX)
