# Supplementary material for: Machine learning approaches for predicting preventive maintenance costs of expressways in Xinjiang
Source: PLoS One. 2026 Jun 16;21(6):e0349595. doi: 10.1371/journal.pone.0349595 (PMC13271440; doi:10.1371/journal.pone.0349595)
Supplement: S1 Table — The raw data of pavement technical condition indicators, maintenance workload, and costs for multiple expressway sections with preventive maintenance. (DOCX) [file pone.0349595.s001.docx]

**Supplementary Table 1: Raw dataset of preventive maintenance projects.**

Each row represents one independent preventive maintenance project, identified by its expressway, year, and treatment type. The dataset is categorized into three preventive maintenance types: crack filling, surface sealing, and overlay. This structure aligns with the modeling framework described in Sections 2.2 and 2.3.

| ID | Highway | Year | Treatment type | PCI | RQI | RDI | SRI | Maintenance workload （m / m²） | Maintenance costs （CNY (Yuan） |
| --- | --- | --- | --- | --- | --- | --- | --- | --- | --- |
|  | G3014 | 2021 | Crack filling | 85.37 | 93.84 | 98.12 | 98.39 | 420 | 6821 |
|  | G30 | 2021 | Crack filling | 83.13 | 93.73 | 94.78 | 97.57 | 27484 | 747362 |
|  | G3012 | 2021 | Crack filling | 84.99 | 93.85 | 93.32 | 97.65 | 27484 | 748257 |
|  | G3013 | 2021 | Crack filling | 91.31 | 81.36 | 83.46 | 87.84 | 19660 | 162938 |
|  | S16 | 2021 | Crack filling | 85.85 | 90.82 | 93.93 | 92.81 | 10317 | 16811 |
|  | G7 | 2021 | Crack filling | 81.64 | 94.81 | 94.54 | 94.97 | 8776 | 65812 |
|  | G30 | 2021 | Crack filling | 86.65 | 92.03 | 89.66 | 90.3 | 36678 | 107722 |
|  | G3012 | 2021 | Crack filling | 86.53 | 93.64 | 91.33 | 97.61 | 190 | 5173 |
|  | G7 | 2022 | Crack filling | 82.47 | 94.41 | 93.84 | 95.31 | 19739 | 355030.74 |
|  | G30 | 2022 | Crack filling | 86.84 | 93.76 | 92.57 | 94.81 | 16204 | 236219.136 |
|  | G30 | 2022 | Crack filling | 86.79 | 93.03 | 86.58 | 89.1 | 1313.65 | 4330.353 |
|  | G30 | 2022 | Crack filling | 80.36 | 93.7 | 93.36 | 95.24 | 14143.09 | 46149.4 |
|  | G30 | 2022 | Crack filling | 86.69 | 93.1 | 91.3 | 91.37 | 5712.69 | 19606.337 |
|  | G3012 | 2022 | Crack filling | 87.31 | 94.2 | 92.56 | 96.81 | 104495 | 365915.87 |
|  | G3012 | 2022 | Crack filling | 87.64 | 92.75 | 90.97 | 95.79 | 41550 | 143841.836 |
|  | G3016 | 2022 | Crack filling | 84.35 | 93.29 | 86.89 | 90.58 | 16245.69 | 54163.053 |
|  | S11 | 2022 | Crack filling | 86.67 | 94.24 | 94.21 | 95.85 | 46000 | 321269.41 |
|  | S12 | 2022 | Crack filling | 83.24 | 93.48 | 94.26 | 94.35 | 6966.08 | 22528.667 |
|  | S16 | 2022 | Crack filling | 83.84 | 93.2 | 96.47 | 88.82 | 49529 | 173548.338 |
|  | G3003 | 2022 | Crack filling | 90.75 | 85.68 | 87.15 | 87.9 | 31343.85 | 596560.984 |
|  | G3015 | 2022 | Crack filling | 87.63 | 94.47 | 87.2 | 88.69 | 11537.1 | 225124.21 |
|  | G3014 | 2022 | Crack filling | 86.69 | 92.36 | 94.45 | 96.47 | 565 | 10826.36 |
|  | G3014 | 2022 | Crack filling | 86.24 | 93.36 | 92.45 | 94.9 | 28393.7 | 583922.64 |
|  | G3014 | 2022 | Crack filling | 82.85 | 93.82 | 92.93 | 95.81 | 21687.79 | 449789.48 |
|  | G30 | 2022 | Crack filling | 85.97 | 92.1 | 93.41 | 94.45 | 49885 | 641488.61 |
|  | G30 | 2022 | Crack filling | 87.01 | 94.12 | 93.16 | 95.14 | 42626.6 | 545108.25 |
|  | G7 | 2022 | Crack filling | 80.64 | 95.42 | 94.84 | 95.63 | 333108.2 | 6440026 |
|  | G30 | 2022 | Crack filling | 87.26 | 93.47 | 91.89 | 93.62 | 49178 | 101307 |
|  | S11 | 2022 | Crack filling | 83.67 | 85.93 | 81.16 | 81.01 | 258112 | 4460062 |
|  | G3014 | 2022 | Crack filling | 83.2 | 94.83 | 93.74 | 98.31 | 111363 | 544975 |
|  | G3015 | 2022 | Crack filling | 85.78 | 93.76 | 93.21 | 97.1 | 130558 | 601672 |
|  | G30 | 2022 | Crack filling | 84.37 | 92.68 | 96.79 | 98.39 | 63553 | 1055409 |
|  | G30 | 2022 | Crack filling | 85.07 | 92.93 | 97.57 | 96.78 | 17653 | 284932.25 |
|  | G3016 | 2022 | Crack filling | 84.53 | 93.24 | 95.25 | 98.57 | 2840 | 47072 |
|  | G3012 | 2022 | Crack filling | 88.31 | 93.68 | 94.78 | 95.46 | 24177 | 967493.57 |
|  | G3012 | 2022 | Crack filling | 83.52 | 95.62 | 94.67 | 97.62 | 202017.5 | 7788292.602 |
|  | G3012 | 2022 | Crack filling | 85.1 | 92.68 | 93.55 | 97.24 | 52340 | 2046378.906 |
|  | G3012 | 2022 | Crack filling | 84.52 | 93.94 | 94.17 | 96.59 | 7672 | 293310.28 |
|  | G3012 | 2022 | Crack filling | 84.32 | 94.83 | 92.79 | 94.9 | 56865 | 2239542.96 |
|  | S22 | 2022 | Crack filling | 87.09 | 81.94 | 89.69 | 93.74 | 118010.22 | 2300867 |
|  | G7 | 2022 | Crack filling | 84.54 | 94.46 | 93.57 | 94.34 | 149085.1 | 2928254.33 |
|  | G3012 | 2022 | Crack filling | 85.46 | 93.62 | 94.67 | 97.35 | 51687.1 | 1021408.39 |
|  | G3012 | 2022 | Crack filling | 83.58 | 94.27 | 93.66 | 95.84 | 30695 | 608771.536 |
|  | G3012 | 2022 | Crack filling | 87.59 | 93.35 | 96.21 | 95.85 | 14148 | 291697.564 |
|  | G3012 | 2023 | Crack filling | 86.49 | 93.16 | 92.85 | 95.85 | 62099.6 | 1479249 |
|  | G3012 | 2023 | Crack filling | 84.3 | 91.82 | 90.47 | 94.44 | 46362.9 | 1084482 |
|  | G3012 | 2023 | Crack filling | 84.47 | 89.25 | 92.56 | 91.65 | 39275.5 | 877438 |
|  | G3012 | 2023 | Crack filling | 83.58 | 92.42 | 93.32 | 92.66 | 22415 | 504437 |
|  | G3012 | 2023 | Crack filling | 80.35 | 89.4 | 92.75 | 95.91 | 204525.9 | 4581209 |
|  | G3012 | 2023 | Crack filling | 81.36 | 94.75 | 95.5 | 96.58 | 303654.1 | 7155121 |
|  | G3014 | 2024 | Crack filling | 90.26 | 94.45 | 93.34 | 95.91 | 384402 | 87771506.22 |
|  | S11 | 2024 | Crack filling | 86.88 | 94.99 | 96.37 | 98.87 | 114771 | 2568163.7 |
|  | G3014 | 2024 | Crack filling | 84.61 | 94.39 | 88.93 | 91.33 | 321860 | 7891574.6 |
|  | G3015 | 2024 | Crack filling | 86.39 | 95.22 | 93.59 | 96.18 | 432363 | 9816494.67 |
|  | G3015 | 2024 | Crack filling | 83.51 | 95.97 | 95.24 | 98.78 | 88410 | 1989250.76 |
|  | G30 | 2024 | Crack filling | 83.71 | 93.85 | 93.35 | 94.7 | 152390 | 2518260.56 |
|  | G30 | 2024 | Crack filling | 83.47 | 94.01 | 92.64 | 90.71 | 51141.65 | 952334.12 |
|  | S12 | 2024 | Crack filling | 91.14 | 95.07 | 92.69 | 80.52 | 3618.7 | 57749.28 |
|  | G3014 | 2024 | Crack filling | 89.48 | 95.82 | 91.26 | 96.47 | 321860 | 7891574.6 |
|  | G30 | 2024 | Crack filling | 86.97 | 93.63 | 95.31 | 97.68 | 136977 | 1899676.04 |
|  | G7 | 2024 | Crack filling | 80.42 | 91.57 | 94.63 | 88.7 | 37340 | 514133.98 |
|  | G3003 | 2024 | Crack filling | 80.1 | 92.02 | 94.84 | 97.97 | 13183 | 181578.14 |
|  | G7 | 2024 | Crack filling | 81.76 | 93.86 | 94.81 | 96.8 | 287322 | 4055960.18 |
|  | G30 | 2024 | Crack filling | 81.38 | 93.7 | 95.2 | 97.8 | 122252 | 1555060.53 |
|  | S11 | 2024 | Crack filling | 80.51 | 94.31 | 93.97 | 96.6 | 69612 | 1045792.73 |
|  | S22 | 2024 | Crack filling | 81.48 | 94.77 | 96.1 | 95.69 | 31780.2 | 421895.88 |
|  | G3012 | 2024 | Crack filling | 85.16 | 94.51 | 97.36 | 94.55 | 129352.3 | 1660729.63 |
|  | G30 | 2024 | Crack filling | 88.88 | 95.49 | 96.44 | 98.08 | 245920.3 | 3248740.16 |
|  | G3012 | 2024 | Crack filling | 90.05 | 93.18 | 96.85 | 95.76 | 31479.78 | 445781.7 |
|  | G3012 | 2024 | Crack filling | 86.87 | 95.33 | 98.42 | 96.21 | 16719.45 | 237910.35 |
|  | S16 | 2024 | Crack filling | 85.77 | 90.83 | 92.74 | 99.12 | 32722.6 | 691863.08 |
|  | G3012 | 2024 | Crack filling | 85.2 | 94.5 | 96.7 | 96.85 | 42223.6 | 596207.74 |
|  | G3013 | 2024 | Crack filling | 85.5 | 93.2 | 96.4 | 97.7 | 74526 | 1446649.56 |
|  | G3012 | 2024 | Crack filling | 80.26 | 93.79 | 97.5 | 98 | 89720.2 | 496604 |
|  | G3014 | 2025 | Crack filling | 89.8 | 95.09 | 93.68 | 96.72 | 237153 | 6781644.94 |
|  | S11 | 2025 | Crack filling | 87 | 95.15 | 95.9 | 98.67 | 75015 | 1670550.57 |
|  | G3014 | 2025 | Crack filling | 86 | 94.4 | 92.4 | 89.4 | 6828 | 153689.44 |
|  | G30 | 2025 | Crack filling | 80.4 | 94.88 | 93.71 | 96.15 | 12990.181 | 163862.93 |
|  | G3014 | 2025 | Crack filling | 90.31 | 94.34 | 85.96 | 81.81 | 23511 | 427325.62 |
|  | G3016 | 2025 | Crack filling | 87.05 | 94.44 | 93.42 | 86.31 | 11250.6 | 155097.63 |
|  | S12 | 2025 | Crack filling | 89.97 | 95.03 | 93.67 | 82.51 | 10952.8 | 184408.19 |
|  | G30 | 2025 | Crack filling | 80.2 | 92.31 | 94.79 | 95.6 | 44025 | 612379.35 |
|  | G7 | 2025 | Crack filling | 80.09 | 93.58 | 95.24 | 89.7 | 142685 | 1965374.42 |
|  | G7 | 2025 | Crack filling | 80.14 | 94.95 | 96.38 | 97.7 | 254390 | 3549182.86 |
|  | G30 | 2025 | Crack filling | 80.46 | 95.1 | 96.2 | 97.8 | 136652 | 2065487.73 |
|  | S11 | 2025 | Crack filling | 86.38 | 97.11 | 95.93 | 97.3 | 8088 | 62104.08 |
|  | S22 | 2025 | Crack filling | 85.19 | 95.33 | 94.73 | 96.53 | 8147.9 | 101305.25 |
|  | G3012 | 2025 | Crack filling | 82.33 | 94.4 | 95.25 | 93.61 | 49828.7 | 654307.69 |
|  | G30 | 2025 | Crack filling | 88.19 | 94.46 | 96.84 | 96.23 | 57596.6 | 760087.67 |
|  | G3012 | 2025 | Crack filling | 80.1 | 94.23 | 94.98 | 94.24 | 16933.66 | 239511.79 |
|  | G3012 | 2025 | Crack filling | 88.67 | 95.31 | 96.11 | 97.1 | 149998.81 | 2134416.46 |
|  | G3012 | 2025 | Crack filling | 85.78 | 92.21 | 94.78 | 97.07 | 1067.55 | 23695.11 |
|  | S13 | 2025 | Crack filling | 81.16 | 95.73 | 96.45 | 98.75 | 147073.9 | 2013614 |
|  | G3012 | 2025 | Crack filling | 88.84 | 90.18 | 96.69 | 98.48 | 65453.87 | 1272850.81 |
|  | G3012 | 2025 | Crack filling | 80.46 | 93.56 | 97.35 | 97.14 | 34181.85 | 189185 |
|  | G30 | 2021 | Surface Sealing | 83.13 | 93.73 | 94.78 | 97.57 | 200000 | 4010526 |
|  | G3012 | 2021 | Surface Sealing | 84.99 | 93.85 | 93.32 | 97.65 | 232280 | 3770161 |
|  | G30 | 2021 | Surface Sealing | 85.97 | 92.1 | 93.41 | 94.45 | 318899 | 5061840.518 |
|  | G30 | 2021 | Surface Sealing | 87.01 | 94.12 | 93.16 | 95.14 | 206687.3 | 3257727.19 |
|  | G3012 | 2021 | Surface Sealing | 85.46 | 93.62 | 94.67 | 97.35 | 644190 | 12460478.54 |
|  | G3012 | 2021 | Surface Sealing | 83.58 | 94.27 | 93.66 | 95.84 | 315980 | 5935921.009 |
|  | G7 | 2021 | Surface Sealing | 81.64 | 94.81 | 94.54 | 94.97 | 132000 | 718354 |
|  | G30 | 2021 | Surface Sealing | 86.65 | 92.03 | 89.66 | 90.3 | 141867 | 1344435 |
|  | G3014 | 2021 | Surface Sealing | 82.85 | 93.82 | 92.93 | 95.81 | 115804.8 | 1974393.74 |
|  | G7 | 2021 | Surface Sealing | 82.47 | 94.41 | 93.84 | 95.31 | 202670 | 3264485.18 |
|  | G30 | 2021 | Surface Sealing | 86.84 | 93.76 | 92.57 | 94.81 | 102644 | 1554206.896 |
|  | G30 | 2021 | Surface Sealing | 86.79 | 93.03 | 86.58 | 89.1 | 175680 | 2724254.784 |
|  | G30 | 2021 | Surface Sealing | 80.36 | 93.7 | 93.36 | 95.24 | 48160 | 728821.592 |
|  | G30 | 2021 | Surface Sealing | 81.64 | 92.74 | 67.3 | 86.37 | 96320 | 1582010.623 |
|  | G3012 | 2021 | Surface Sealing | 87.31 | 94.2 | 92.56 | 96.81 | 375000 | 7431979.662 |
|  | G3012 | 2021 | Surface Sealing | 87.64 | 92.75 | 90.97 | 95.79 | 72000 | 1376333.376 |
|  | S16 | 2021 | Surface Sealing | 83.84 | 93.2 | 96.47 | 88.82 | 112000 | 2075472.42 |
|  | G3015 | 2021 | Surface Sealing | 87.63 | 94.47 | 87.2 | 88.69 | 35924.7 | 565012.4 |
|  | G3014 | 2021 | Surface Sealing | 86.24 | 93.36 | 92.45 | 94.9 | 15800 | 239431.6 |
|  | G7 | 2021 | Surface Sealing | 80.64 | 95.42 | 94.84 | 95.63 | 49144 | 859040 |
|  | G3015 | 2021 | Surface Sealing | 85.78 | 93.76 | 93.21 | 97.1 | 33600 | 658829 |
|  | G30 | 2021 | Surface Sealing | 83.13 | 93.73 | 94.78 | 97.57 | 224352 | 4980702 |
|  | G3012 | 2021 | Surface Sealing | 84.99 | 93.85 | 93.32 | 97.65 | 238790 | 5325556 |
|  | G7 | 2021 | Surface Sealing | 81.64 | 94.81 | 94.54 | 94.97 | 323588 | 6955354 |
|  | G30 | 2021 | Surface Sealing | 86.65 | 92.03 | 89.66 | 90.3 | 141867 | 3297073 |
|  | G30 | 2021 | Surface Sealing | 86.65 | 92.03 | 87.66 | 88.3 | 185404 | 4308898 |
|  | G3012 | 2021 | Surface Sealing | 86.53 | 93.64 | 91.33 | 97.61 | 115500 | 2892664 |
|  | G3013 | 2021 | Surface Sealing | 91.31 | 81.36 | 83.46 | 87.84 | 610560 | 25369144 |
|  | S16 | 2021 | Surface Sealing | 85.85 | 90.82 | 93.93 | 92.81 | 288000 | 11171025 |
|  | G30 | 2021 | Surface Sealing | 87.01 | 94.12 | 93.16 | 95.14 | 191340.3 | 3752566.48 |
|  | G30 | 2022 | Surface Sealing | 85.97 | 92.1 | 93.41 | 94.45 | 286378.5 | 5701803.792 |
|  | G7 | 2022 | Surface Sealing | 82.47 | 94.41 | 93.84 | 95.31 | 201360 | 4865386.49 |
|  | G30 | 2022 | Surface Sealing | 86.84 | 93.76 | 92.57 | 94.81 | 102644 | 2260164.386 |
|  | G30 | 2022 | Surface Sealing | 86.79 | 93.03 | 86.58 | 89.1 | 175680 | 4015479.331 |
|  | G30 | 2022 | Surface Sealing | 80.36 | 93.7 | 93.36 | 95.24 | 48160 | 1085832.843 |
|  | G30 | 2022 | Surface Sealing | 86.69 | 93.1 | 91.3 | 91.37 | 96320 | 2334487.981 |
|  | G3012 | 2022 | Surface Sealing | 87.31 | 94.2 | 92.56 | 96.81 | 1065200 | 26572828.25 |
|  | G3012 | 2022 | Surface Sealing | 87.64 | 92.75 | 90.97 | 95.79 | 264000 | 7142770.459 |
|  | G3016 | 2022 | Surface Sealing | 84.35 | 93.29 | 86.89 | 90.58 | 156840 | 3539703.73 |
|  | S11 | 2022 | Surface Sealing | 86.67 | 94.24 | 94.21 | 95.85 | 172780 | 2705890.73 |
|  | S12 | 2022 | Surface Sealing | 83.24 | 93.48 | 94.26 | 94.35 | 115360 | 2522461.92 |
|  | S16 | 2022 | Surface Sealing | 83.84 | 93.2 | 96.47 | 88.82 | 249600 | 6042326.99 |
|  | G3012 | 2022 | Surface Sealing | 85.46 | 93.62 | 94.67 | 97.35 | 644190 | 13736666.52 |
|  | G3012 | 2022 | Surface Sealing | 83.58 | 94.27 | 93.66 | 95.84 | 315980 | 6772122.816 |
|  | G3014 | 2022 | Surface Sealing | 86.69 | 92.36 | 94.45 | 96.47 | 14176 | 232944.05 |
|  | G3014 | 2022 | Surface Sealing | 86.24 | 93.36 | 92.45 | 94.9 | 42648.7 | 717814.47 |
|  | G3014 | 2022 | Surface Sealing | 83.2 | 94.83 | 93.74 | 98.31 | 336000 | 7845794 |
|  | G3015 | 2022 | Surface Sealing | 85.78 | 93.76 | 93.21 | 97.1 | 296000 | 6711200 |
|  | G3014 | 2022 | Surface Sealing | 82.85 | 93.82 | 92.93 | 95.81 | 164627.2 | 5852230.54 |
|  | G3015 | 2022 | Surface Sealing | 85.78 | 93.76 | 93.21 | 97.1 | 121963.3 | 4029363.79 |
|  | G3014 | 2022 | Surface Sealing | 86.24 | 93.36 | 92.45 | 94.9 | 331816 | 10613055.97 |
|  | G3014 | 2022 | Surface Sealing | 82.85 | 93.82 | 92.93 | 95.81 | 58115.5 | 330216.18 |
|  | G3015 | 2022 | Surface Sealing | 85.78 | 93.76 | 93.21 | 97.1 | 9435.25 | 50310.82 |
|  | G3014 | 2022 | Surface Sealing | 86.24 | 93.36 | 92.45 | 94.9 | 11773.5 | 64023.9 |
|  | G3014 | 2022 | Surface Sealing | 85.37 | 93.84 | 98.12 | 98.39 | 236250 | 5553471 |
|  | G3003 | 2022 | Surface Sealing | 90.75 | 85.68 | 87.15 | 87.9 | 321080 | 5493740.594 |
|  | G3012 | 2022 | Surface Sealing | 87.59 | 93.35 | 96.21 | 95.85 | 64000 | 1144237.921 |
|  | G3003 | 2022 | Surface Sealing | 90.75 | 85.68 | 87.15 | 87.9 | 321080 | 6300424.329 |
|  | G3012 | 2022 | Surface Sealing | 87.59 | 93.35 | 96.21 | 95.85 | 64800 | 1336286.066 |
|  | G3012 | 2022 | Surface Sealing | 86.53 | 93.64 | 91.33 | 97.61 | 115500 | 2892664 |
|  | S11 | 2022 | Surface Sealing | 86.67 | 94.24 | 94.21 | 95.85 | 172780 | 4126192.48 |
|  | G3014 | 2023 | Surface Sealing | 83.2 | 94.83 | 93.74 | 98.31 | 24000 | 481159 |
|  | G3012 | 2023 | Surface Sealing | 83.52 | 95.62 | 94.67 | 97.62 | 25080 | 549818.06 |
|  | G3012 | 2023 | Surface Sealing | 86.49 | 93.16 | 92.85 | 95.85 | 261746 | 8988000 |
|  | G30 | 2024 | Surface Sealing | 83.71 | 93.85 | 93.35 | 94.7 | 685696.6 | 12827765.68 |
|  | G30 | 2024 | Surface Sealing | 83.47 | 94.01 | 92.64 | 90.71 | 96000 | 1791984.55 |
|  | G3012 | 2024 | Surface Sealing | 85.16 | 94.51 | 97.36 | 94.55 | 123152.6 | 2436955.88 |
|  | S16 | 2024 | Surface Sealing | 85.77 | 90.83 | 92.74 | 99.12 | 255760 | 11499561.07 |
|  | G3012 | 2024 | Surface Sealing | 85.2 | 94.5 | 96.7 | 96.85 | 295790 | 12851106.69 |
|  | G3013 | 2024 | Surface Sealing | 85.5 | 93.2 | 96.4 | 97.7 | 619200 | 28895001.24 |
|  | G3012 | 2024 | Surface Sealing | 80.26 | 93.79 | 97.5 | 98 | 54060 | 2264474 |
|  | G30 | 2025 | Surface Sealing | 80.4 | 94.88 | 93.71 | 96.15 | 294500 | 5524212.06 |
|  | G3014 | 2025 | Surface Sealing | 90.31 | 94.34 | 85.96 | 81.81 | 8000 | 149438.88 |
|  | S13 | 2025 | Surface Sealing | 81.16 | 95.73 | 96.45 | 98.75 | 173577 | 3491624 |
|  | G3012 | 2025 | Surface Sealing | 88.84 | 90.18 | 96.69 | 98.48 | 232572.75 | 4745430.98 |
|  | G3012 | 2022 | Overlay | 85.46 | 93.62 | 94.67 | 97.35 | 56000 | 2800000 |
|  | G3014 | 2022 | Overlay | 83.2 | 94.83 | 93.74 | 98.31 | 305215 | 14478580 |
|  | G3015 | 2022 | Overlay | 85.78 | 93.76 | 93.21 | 97.1 | 151488 | 8227773 |
|  | G3012 | 2022 | Overlay | 83.52 | 95.62 | 94.67 | 97.62 | 598000 | 9559962.054 |
|  | G3012 | 2022 | Overlay | 85.1 | 92.68 | 93.55 | 97.24 | 287845 | 7301393.41 |
|  | G30 | 2022 | Overlay | 87.94 | 94 | 92.1 | 93.7 | 230448 | 23676192 |
|  | G30 | 2022 | Overlay | 85.68 | 92.16 | 93.57 | 96.91 | 72880 | 3935520 |
|  | G3012 | 2022 | Overlay | 88.31 | 93.68 | 94.78 | 95.46 | 312000 | 16284527 |
|  | G3012 | 2022 | Overlay | 85.1 | 92.68 | 93.55 | 97.24 | 150811 | 7301393.4 |
|  | G3012 | 2022 | Overlay | 84.52 | 93.94 | 94.17 | 96.59 | 98700 | 5173197.1 |
|  | G3012 | 2022 | Overlay | 84.32 | 94.83 | 92.79 | 94.9 | 632000 | 32133475 |
|  | G30 | 2022 | Overlay | 83.68 | 81.46 | 85.82 | 96.85 | 96000 | 4320000 |
|  | G7 | 2023 | Overlay | 80.64 | 95.42 | 94.84 | 95.63 | 1203834 | 66333335 |
|  | S11 | 2023 | Overlay | 83.67 | 85.93 | 81.16 | 81.01 | 718335 | 39609709 |
|  | G30 | 2023 | Overlay | 87.26 | 93.47 | 91.89 | 93.62 | 195641 | 9964284 |
|  | S22 | 2023 | Overlay | 87.09 | 81.94 | 89.69 | 93.74 | 415780.4 | 8905551.09 |
|  | G7 | 2023 | Overlay | 84.54 | 94.46 | 93.57 | 94.34 | 354300 | 7361240.5 |
|  | S22 | 2023 | Overlay | 87.09 | 81.94 | 89.69 | 93.74 | 415780.4 | 20562506.36 |
|  | G7 | 2023 | Overlay | 84.54 | 94.46 | 93.57 | 94.34 | 354300 | 17788197.13 |
|  | G3012 | 2023 | Overlay | 86.49 | 93.16 | 92.85 | 95.85 | 206051 | 9308597 |
|  | G3012 | 2023 | Overlay | 86.49 | 93.16 | 92.85 | 95.85 | 39413 | 1335407 |
|  | G3012 | 2023 | Overlay | 84.3 | 91.82 | 90.47 | 94.44 | 302366 | 14973270 |
|  | G3012 | 2023 | Overlay | 84.47 | 89.25 | 92.56 | 91.65 | 387157 | 18415453 |
|  | G3012 | 2023 | Overlay | 83.58 | 92.42 | 93.32 | 92.66 | 133258 | 6992518 |
|  | G3012 | 2023 | Overlay | 80.35 | 89.4 | 92.75 | 95.91 | 23200 | 1126424 |
|  | G3012 | 2023 | Overlay | 81.36 | 94.75 | 95.5 | 96.58 | 669832 | 32273301 |
|  | G3014 | 2024 | Overlay | 90.26 | 94.45 | 93.34 | 95.91 | 1058808 | 48822061.49 |
|  | S11 | 2024 | Overlay | 86.88 | 94.99 | 96.37 | 98.87 | 377872 | 17365452.5 |
|  | G3014 | 2024 | Overlay | 84.61 | 94.39 | 88.93 | 91.33 | 737412 | 36552755.89 |
|  | G3015 | 2024 | Overlay | 86.39 | 95.22 | 93.59 | 96.18 | 1143388 | 53708079.8 |
|  | G3015 | 2024 | Overlay | 86.39 | 95.22 | 93.59 | 96.18 | 59344 | 3189804.17 |
|  | G30 | 2024 | Overlay | 83.71 | 93.85 | 93.35 | 94.7 | 1287952.6 | 53164914.23 |
|  | G30 | 2024 | Overlay | 83.47 | 94.01 | 92.64 | 90.71 | 622517 | 27137862.44 |
|  | S12 | 2024 | Overlay | 91.14 | 95.07 | 92.69 | 80.52 | 112000 | 5265048.07 |
|  | G3014 | 2024 | Overlay | 89.48 | 95.82 | 91.26 | 96.47 | 737412 | 36552755.88 |
|  | G30 | 2024 | Overlay | 86.97 | 93.63 | 95.31 | 97.68 | 982979 | 40888641.84 |
|  | G7 | 2024 | Overlay | 80.42 | 91.57 | 94.63 | 88.7 | 173188 | 8794683.59 |
|  | G3003 | 2024 | Overlay | 80.1 | 92.02 | 94.84 | 97.97 | 107748 | 4402806.47 |
|  | G7 | 2024 | Overlay | 81.76 | 93.86 | 94.81 | 96.8 | 1091430 | 49984529.16 |
|  | G30 | 2024 | Overlay | 81.38 | 93.7 | 95.2 | 97.8 | 676270 | 29620761.5 |
|  | S11 | 2024 | Overlay | 80.51 | 94.31 | 93.97 | 96.6 | 167680 | 8145572.81 |
|  | S22 | 2024 | Overlay | 81.48 | 94.77 | 96.1 | 95.69 | 123600 | 6913171.23 |
|  | G3012 | 2024 | Overlay | 85.16 | 94.51 | 97.36 | 94.55 | 439235.8 | 24192940.53 |
|  | G30 | 2024 | Overlay | 88.88 | 95.49 | 96.44 | 98.08 | 756044.8 | 41364093.36 |
|  | G30 | 2024 | Overlay | 88.88 | 95.49 | 96.44 | 98.08 | 67644.8 | 3423490.41 |
|  | G3012 | 2024 | Overlay | 90.05 | 93.18 | 96.85 | 95.76 | 231648.96 | 8503029.66 |
|  | G3012 | 2024 | Overlay | 86.87 | 95.33 | 98.42 | 96.21 | 273744 | 10614814.34 |
|  | S16 | 2024 | Overlay | 85.77 | 90.83 | 92.74 | 99.12 | 102680 | 5637123.97 |
|  | G3012 | 2024 | Overlay | 85.2 | 94.5 | 96.7 | 96.85 | 103920 | 5752670.68 |
|  | G3014 | 2025 | Overlay | 89.8 | 95.09 | 93.68 | 96.72 | 550208 | 26898081.45 |
|  | S11 | 2025 | Overlay | 87 | 95.15 | 95.9 | 98.67 | 166112 | 8912103.4 |
|  | G3014 | 2025 | Overlay | 86 | 94.4 | 92.4 | 89.4 | 19780 | 952692.92 |
|  | G3015 | 2025 | Overlay | 83.51 | 95.97 | 95.24 | 98.78 | 272320 | 12624474.45 |
|  | G30 | 2025 | Overlay | 80.4 | 94.88 | 93.71 | 96.15 | 294500 | 11974744.45 |
|  | G3014 | 2025 | Overlay | 90.31 | 94.34 | 85.96 | 81.81 | 429900.75 | 17582347.83 |
|  | G3016 | 2025 | Overlay | 87.05 | 94.44 | 93.42 | 86.31 | 160000 | 7555331.02 |
|  | S12 | 2025 | Overlay | 89.97 | 95.03 | 93.67 | 82.51 | 280000 | 13109923.07 |
|  | G30 | 2025 | Overlay | 80.2 | 92.31 | 94.79 | 95.6 | 281121 | 11855336.79 |
|  | G7 | 2025 | Overlay | 80.09 | 93.58 | 95.24 | 89.7 | 585102 | 29946626.95 |
|  | G7 | 2025 | Overlay | 80.14 | 94.95 | 96.38 | 97.7 | 1313532 | 59104202.46 |
|  | G30 | 2025 | Overlay | 80.46 | 95.1 | 96.2 | 97.8 | 925270 | 40528235.36 |
|  | S11 | 2025 | Overlay | 86.38 | 97.11 | 95.93 | 97.3 | 250336 | 12631971.7 |
|  | G3012 | 2025 | Overlay | 82.33 | 94.4 | 95.25 | 93.61 | 150861.3 | 8256473.65 |
|  | G30 | 2025 | Overlay | 88.19 | 94.46 | 96.84 | 96.23 | 208644.8 | 11242812.29 |
|  | G3012 | 2025 | Overlay | 80.1 | 94.23 | 94.98 | 94.24 | 107817.44 | 3758681 |
|  | G3012 | 2025 | Overlay | 88.67 | 95.31 | 96.11 | 97.1 | 1075336 | 41675560 |
|  | G3012 | 2025 | Overlay | 85.78 | 92.21 | 94.78 | 97.07 | 111660 | 5387824.94 |
|  | G3012 | 2025 | Overlay | 88.84 | 90.18 | 96.69 | 98.48 | 40000 | 943145.14 |
|  | G3012 | 2025 | Overlay | 80.46 | 93.56 | 97.35 | 97.14 | 16740 | 705458 |
