# Supplementary material for: Machine learning approaches for predicting preventive maintenance costs of expressways in Xinjiang
Source: PLoS One. 2026 Jun 16;21(6):e0349595. doi: 10.1371/journal.pone.0349595 (PMC13271440; doi:10.1371/journal.pone.0349595)
Supplement: S2 Table — The metadata and descriptive information of all variables included in the raw dataset and predictive models, including definitions, units, value ranges. (DOCX) [file pone.0349595.s002.docx]

**Supplementary Table 2: Metadata and descriptive statistics of model variables.**

This table provides a comprehensive overview of all input, output, and categorical variables used in the predictive modeling framework. It includes variable definitions, measurement units, value ranges, and contextual notes to ensure data transparency and reproducibility. Each variable is categorized by its role in the analysis (Input, Output, or Categorical), with detailed descriptions.

| Variable Name | Full Name | Variable Type | Unit | Value Range | Description | Notes |
| --- | --- | --- | --- | --- | --- | --- |
| PCI | Pavement Condition Index | Input | dimensionless | 0–100 | Represents the overall pavement surface condition based on distress severity and extent | Higher values indicate better condition |
| RQI | Ride Quality Index | Input | dimensionless | 0–100 | Reflects pavement ride quality derived from surface roughness (related to IRI) | Higher values indicate smoother pavement |
| RDI | Rutting Depth Index | Input | dimensionless | 0–100 | Indicates the severity of rutting deformation on pavement surface | Higher values indicate less rutting |
| SRI | Skid Resistance Index | Input | dimensionless | 0–100 | Represents pavement skid resistance performance related to surface friction | Higher values indicate better skid resistance |
| Workload | Maintenance workload | Input | m (crack filling) or m² (surface sealing & overlay) | Project-dependent | Quantity of maintenance work performed for a given project | Value RaUnit depends on maintenance typenge |
| Cost | Maintenance cost | Output | CNY (Yuan) | Project-dependent | Total cost of preventive maintenance for each project | Includes labor, material, and equipment costs |
| Maintenance type | Maintenance type | Categorical | — | Crack filling,  Surface sealing,  Overlay | Type of preventive maintenance treatment applied | Used to group dataset for different models |
