# Supplementary material for: Machine learning approaches for predicting preventive maintenance costs of expressways in Xinjiang
Source: PLoS One. 2026 Jun 16;21(6):e0349595. doi: 10.1371/journal.pone.0349595 (PMC13271440; doi:10.1371/journal.pone.0349595)
Supplement: S3 Table — The 10-fold nested cross-validation outcomes for six predictive models, showing fold-specific performance metrics. (DOCX) [file pone.0349595.s003.docx]

**Supplementary Table 3: Nested cross-validation results for predictive models.**

The 10-fold nested cross-validation outcomes for six predictive models, showing fold-specific performance metrics.

**For crack filling:**

XGBoost model ten-fold nested cross-validation experiment results.

| Ford | MAE | MAPE (%) | MSE | RMSE |
| --- | --- | --- | --- | --- |
| 1 | 0.0767 | 6.1699 | 0.0104 | 0.1019 |
| 2 | 0.0569 | 4.0358 | 0.0059 | 0.0768 |
| 3 | 0.0680 | 4.9749 | 0.0080 | 0.0892 |
| 4 | 0.0621 | 4.6048 | 0.0060 | 0.0775 |
| 5 | 0.0787 | 5.8146 | 0.0110 | 0.1051 |
| 6 | 0.0828 | 5.8071 | 0.0096 | 0.0981 |
| 7 | 0.0699 | 4.1369 | 0.0113 | 0.1062 |
| 8 | 0.0593 | 4.0720 | 0.0053 | 0.0728 |
| 9 | 0.0648 | 4.5884 | 0.0071 | 0.0843 |
| 10 | 0.0589 | 3.9869 | 0.0051 | 0.0712 |

FOA-XGBoost model ten-fold nested cross-validation experiment results.

| Ford | MAE | MAPE (%) | MSE | RMSE |
| --- | --- | --- | --- | --- |
| 1 | 0.0772 | 6.3739 | 0.0112 | 0.1056 |
| 2 | 0.0553 | 3.9849 | 0.0058 | 0.0759 |
| 3 | 0.0703 | 5.0619 | 0.0080 | 0.0893 |
| 4 | 0.0673 | 4.9270 | 0.0077 | 0.0876 |
| 5 | 0.0782 | 5.6720 | 0.0097 | 0.0986 |
| 6 | 0.0651 | 4.5092 | 0.0061 | 0.0781 |
| 7 | 0.0584 | 3.4050 | 0.0105 | 0.1027 |
| 8 | 0.0439 | 2.9778 | 0.0026 | 0.0506 |
| 9 | 0.0653 | 4.6023 | 0.0071 | 0.0843 |
| 10 | 0.0533 | 3.5730 | 0.0044 | 0.0661 |

**For surface sealing:**

RF model ten-fold nested cross-validation experiment results.

| Ford | MAE | MAPE (%) | MSE | RMSE |
| --- | --- | --- | --- | --- |
| 1 | 0.0329 | 2.0115 | 0.0013 | 0.0357 |
| 2 | 0.0448 | 2.7001 | 0.0033 | 0.0576 |
| 3 | 0.0468 | 3.1153 | 0.0031 | 0.0558 |
| 4 | 0.0618 | 4.5281 | 0.0062 | 0.0784 |
| 5 | 0.0219 | 1.3540 | 0.0006 | 0.0254 |
| 6 | 0.0927 | 5.9899 | 0.0120 | 0.1095 |
| 7 | 0.0546 | 3.2564 | 0.0043 | 0.0657 |
| 8 | 0.0553 | 3.3419 | 0.0065 | 0.0805 |
| 9 | 0.0524 | 3.9070 | 0.0046 | 0.0678 |
| 10 | 0.0733 | 4.9032 | 0.0105 | 0.1024 |

HOA-RF model ten-fold nested cross-validation experiment results.

| Ford | MAE | MAPE (%) | MSE | RMSE |
| --- | --- | --- | --- | --- |
| 1 | 0.0325 | 1.9669 | 0.0013 | 0.0357 |
| 2 | 0.0433 | 2.5671 | 0.0035 | 0.0589 |
| 3 | 0.0361 | 2.3287 | 0.0018 | 0.0419 |
| 4 | 0.0615 | 4.5327 | 0.0068 | 0.0826 |
| 5 | 0.0250 | 1.5425 | 0.0008 | 0.0284 |
| 6 | 0.0908 | 5.8535 | 0.0118 | 0.1087 |
| 7 | 0.0437 | 2.5974 | 0.0031 | 0.0553 |
| 8 | 0.0542 | 3.2581 | 0.0057 | 0.0754 |
| 9 | 0.0660 | 5.0243 | 0.0090 | 0.0946 |
| 10 | 0.0705 | 4.7405 | 0.0099 | 0.0993 |

**For overlay:**

BPNN model ten-fold nested cross-validation experiment results.

| Ford | MAE | MAPE (%) | MSE | RMSE |
| --- | --- | --- | --- | --- |
| 1 | 0.0399 | 2.5324 | 0.0029 | 0.0539 |
| 2 | 0.1042 | 5.9837 | 0.0146 | 0.1210 |
| 3 | 0.0519 | 3.7146 | 0.0058 | 0.0764 |
| 4 | 0.0477 | 2.7626 | 0.0027 | 0.0517 |
| 5 | 0.0360 | 2.0680 | 0.0016 | 0.0404 |
| 6 | 0.0536 | 3.3196 | 0.0077 | 0.0880 |
| 7 | 0.0719 | 5.2825 | 0.0094 | 0.0968 |
| 8 | 0.0714 | 5.0309 | 0.0082 | 0.0905 |
| 9 | 0.0676 | 4.1544 | 0.0065 | 0.0806 |
| 10 | 0.0883 | 7.2275 | 0.0139 | 0.1180 |

PSO-BPNN model ten-fold nested cross-validation experiment results.

| Ford | MAE | MAPE (%) | MSE | RMSE |
| --- | --- | --- | --- | --- |
| 1 | 0.0407 | 2.6322 | 0.0031 | 0.0560 |
| 2 | 0.0540 | 3.1106 | 0.0061 | 0.0783 |
| 3 | 0.0460 | 3.2191 | 0.0057 | 0.0754 |
| 4 | 0.0235 | 1.3768 | 0.0009 | 0.0298 |
| 5 | 0.0198 | 1.1497 | 0.0005 | 0.0232 |
| 6 | 0.0751 | 4.6821 | 0.0116 | 0.1078 |
| 7 | 0.0750 | 5.4394 | 0.0089 | 0.0944 |
| 8 | 0.0265 | 1.7961 | 0.0008 | 0.0287 |
| 9 | 0.0367 | 2.2263 | 0.0021 | 0.0459 |
| 10 | 0.0241 | 1.9217 | 0.0013 | 0.0363 |
