# Supplementary material for: Machine learning approaches for predicting preventive maintenance costs of expressways in Xinjiang
Source: PLoS One. 2026 Jun 16;21(6):e0349595. doi: 10.1371/journal.pone.0349595 (PMC13271440; doi:10.1371/journal.pone.0349595)
Supplement: Attachment — Submitted filename: Response to Reviewers.pdf [file pone.0349595.s005.pdf]

Response to the Reviewer's Comments

Manuscript number: PONE-D-25-52655

Title: Machine learning approaches for predicting preventive maintenance costs of expressways in Xinjiang

Dear Editors and Reviewers,

We appreciate the opportunity to revise our manuscript titled "Machine learning approaches for predicting preventive maintenance costs of expressways in Xinjiang" and are grateful for the insightful comments provided by the reviewers. Those comments are all valuable and very helpful for revising and improving our paper, and have greatly improved the quality of our work. We have carefully considered all comments and revised the manuscript accordingly. Detailed responses to the reviewers' comments are provided in the response letter, and the corresponding revisions have been clearly marked in the revised manuscript.

In addition, we have addressed the specific requirements raised by the journal, as detailed below.

1. Code sharing compliance

We have reviewed the PLOS ONE guidelines on code sharing and understand the journal's requirement that all author-generated code underpinning the findings of the manuscript should be made available without restrictions upon publication of the work. We confirm that all author-generated code underpinning the findings of this study will be made publicly available upon publication of the manuscript.

2. Funding information

We sincerely apologize for the inconsistency between the grant information previously provided in the "Funding Information" and "Financial Disclosure" sections. We have carefully checked and corrected the funding information. This research was supported only by the following two projects:

Xinjiang Transportation Investment (Group) Co., Ltd. Supported Project (Grant No.: EKXFWCG2024040202);

Xinjiang Natural Science Foundation Project (Grant No.: 2024D01A53).

3. Role of funders

The roles of the funders in this study are as follows: Xinjiang Transportation Investment (Group) Co., Ltd. (Grant No.: EKXFWCG2024040202) participated in the study design, provided guidance on data collection methods, and supported data collection and experimental operations financially. Xinjiang Natural Science Foundation Project (Grant No.: 2024D01A53) provided financial support for manuscript revision and offered general suggestions for improvement of the manuscript.

4. Reviewer-suggested references

We have carefully reviewed the references suggested by the reviewers and evaluated their relevance to our study. Relevant works have been considered and incorporated where appropriate in the revised manuscript.

We have carefully followed the journal’s revision requirements and sincerely hope that the revised manuscript and response letter meet the standards for publication in PLOS ONE.

Thank you very much for your time, consideration, and valuable guidance.

Sincerely,

The Authors

Reviewer #1:

Comment 1: Expand the Dataset and Validate Across Regions

Future work should include datasets from other provinces or countries to test the adaptability of the hybrid models under different environmental and economic conditions. This would increase the model's credibility and relevance for broader infrastructure management applications.

Response 1:

We appreciate this important suggestion. We agree that cross-regional validation would further enhance the generalizability of the proposed models. Due to the limited availability of consistent and high-resolution maintenance cost data across regions, such validation could not be incorporated in the current study. To partially address this concern, we explicitly outlined cross-regional validation as a key direction for future work.

We have added the following statement in the Conclusion section (page 21, lines 700–704):

"*Further integration of expressway maintenance data from regions with more diverse climatic conditions and socioeconomic backgrounds, such as southern China and other countries worldwide, would enable more robust validation and refinement of the hybrid model proposed in this study, thereby enhancing its generalizability and practical utility for infrastructure management in broader contexts.*"

Comment 2: Introduce Temporal and Econometric Analysis

Integrating time-dependent variables, such as inflation-adjusted costs, maintenance frequency, or environmental degradation rates, would enable dynamic forecasting. Incorporating time-series models (e.g., LSTM, Prophet, or hybrid ARIMA-ML frameworks) could enhance predictive precision in long-term maintenance planning.

Response 2:

We agree that incorporating temporal dynamics would improve long-term predictive capability. However, in the current study, the available dataset mainly consists of short-term panel data (2021–2025), which is not sufficient to support the training and validation of complex time-series models. In addition, the primary objective of this study is to perform a cross-sectional comparison of feature engineering and machine learning optimization under different preventive maintenance strategies. We have added a discussion on the integration of temporal models and macroeconomic variables in future work.

To address this important comment, we have added a corresponding discussion in the Conclusion section of the revised manuscript (page 21, lines 704–707):

"Simultaneously, collecting long-term monitoring data and incorporating macroeconomic variables, together with time-series models such as LSTM or ARIMA-based hybrid approaches, would enable dynamic maintenance cost prediction over longer life cycles."

Comment 3: Strengthen Validation to Mitigate Overfitting

The authors are encouraged to employ more advanced validation schemes such as nested cross-validation or holdout testing using independent datasets. Sensitivity analysis and hyperparameter uncertainty quantification would further support claims of model robustness.

Response 3:

We fully agree that rigorous validation and robustness analysis are essential for reducing overfitting risk and improving the reliability of predictive models.

In response to this comment, we have substantially strengthened both the validation framework and the robustness analysis in the revised manuscript. Specifically, we replaced the original 5-fold cross-validation with a 10-fold nested cross-validation scheme, and incorporated single-factor sensitivity analysis to further evaluate model robustness and interpretability. Corresponding revisions were made in both the Materials and methods and Case study sections.

More specifically, the relevant revisions have been incorporated into the following sections of the revised manuscript:

Section 2.5 Model evaluation *(page 12, lines 375–378), where the evaluation framework has been clarified;*

Section 2.5.2 Nested cross-validation *(page 13, lines 396–423), where the nested cross-validation procedure and confidence interval estimation have been added;*

Section 2.7 Influencing factor analysis and sensitivity analysis *(page 15, lines 478–488), where the rationale and procedure of sensitivity analysis are described;*

Section 3.4 Cross-validation experimental results *(pages 18–19, lines 577–611), where the validation results under the revised framework are reported;*

Section 3.5 Results of influencing factor and sensitivity analyses *(pages 19–20, lines 612–649), where the robustness and interpretability results are presented.*

Comment 4: Deepen Ethical and Practical Considerations

Although this research involves infrastructure rather than personal data, ethical and practical implications remain relevant—particularly in algorithmic decision-making that influences public resource allocation. A brief reflection on model transparency, explainability, and decision accountability in automated maintenance systems would be beneficial.

Response 4:

We fully agree with the reviewer, although this study does not involve personal data, ethical and practical considerations remain highly relevant, particularly in the context of algorithmic decision-making for public resource allocation.

In response to this important concern, we have added a dedicated discussion in the Conclusion section of the revised manuscript (page 21, lines 720–727), addressing issues related to model transparency, interpretability, and decision accountability in practical applications. The added text reads:

"In practical applications, additional considerations are also necessary. When

applied to public resource allocation, issues related to transparency, interpretability, and decision accountability should be carefully addressed. The integration of explainable artificial intelligence (XAI) techniques can help clarify the contribution of input features and improve model interpretability. At the same time, the proposed models are intended to serve as decision-support tools rather than fully automated decision-making systems, and human oversight remains essential to ensure responsible and accountable use of maintenance resources."

Comment 5: Expand Discussion on Policy and Economic Integration

The authors should elaborate on how such predictive tools can be institutionalized within existing Pavement Management Systems (PMS) or governmental budget frameworks. Discussing cost–benefit implications or potential policy adoption pathways would elevate the manuscript's practical contribution to infrastructure governance.

Response 5:

We sincerely thank the reviewer for this valuable suggestion. We agree that further elaboration on policy integration and practical implementation would enhance the real-world relevance and applicability of the proposed models.

To address this comment, we have expanded the discussion in the Conclusion section of the revised manuscript (page 21, lines 728–736), where we describe how the proposed predictive framework can be integrated into existing Pavement

Management Systems (PMS) and its potential role in supporting budget planning and decision-making processes. The added text reads:

"From an application perspective, the proposed hybrid models can be integrated into existing Pavement Management Systems (PMS) as a cost prediction module. Once maintenance needs are identified within the PMS, the appropriate model can be selected to generate cost estimates based on key pavement performance indicators. This data-driven approach can support preliminary budget planning, facilitate a transition from reactive to preventive maintenance strategies, and improve cost-effectiveness over the entire lifecycle. Furthermore, such integration provides a pathway for embedding data-driven cost estimation into existing decision-support workflows within infrastructure management agencies."

We sincerely thank the reviewer for recommending these high-quality and relevant references. We have carefully reviewed these studies and incorporated them into the revised manuscript in appropriate sections to strengthen the literature review and methodological grounding of the study.

Specifically, Refs. [27] and [28] have been cited in the Introduction section (page 3, lines 63–65) in support of the following statement:

"Consequently, a growing body of research has focused on integrating metaheuristic algorithms with machine learning models to develop more effective hybrid models [27,28]."

In addition, Ref. [43] has been added in the Materials and methods section (page 14, line 429), Wilcoxon signed-rank test to support the statistical comparison between the optimized hybrid models and their corresponding baseline models. The revised text reads:

"To evaluate whether the performance differences between the optimized hybrid models and their baseline counterparts are statistically significant, the Wilcoxon signed-rank test was conducted based on cross-validation results, where key performance metrics (e.g., MAE and RMSE) obtained from each fold were treated as paired samples for model comparison. Statistical significance was determined at a predefined level of $\alpha = 0.05$ *[43]."*

The newly added references have also been marked in the reference list for ease of checking.

Reviewer #2:

Comment 1: Study design and data description

The manuscript does not clearly explain what each observation represents (e.g., spatial unit, time period, or scenario). Key information on data structure, sample size, and replication is missing, limiting the interpretability of the results.

Response 1:

This is an important point. We agree that a clear description of the study design and dataset structure is essential for improving the interpretability and reproducibility of the results.

In response to this comment, we have substantially revised the manuscript to clarify the definition of observations, dataset organization, and sample distribution. Specifically, we now explicitly define each observation as an individual preventive maintenance project represented by “expressway section × maintenance treatment × year”, and we provide detailed information on the dataset structure, total sample size (239 observations), and the sample sizes for each maintenance category.

We have also revised the Data sources and preprocessing section to clearly describe the dataset organization and its role in the modeling framework, and we explicitly state that the complete raw dataset is provided in S1 Table, with consistent preprocessing procedures applied across all samples. In addition, a fixed random seed has been introduced to enhance the reproducibility of the experiments.

The revised and newly added text is provided below for clarity:

2.2 Data sources and preprocessing

(pages 4–5, lines 144–150)

This study collected data from preventive maintenance projects implemented on 13 expressways in Xinjiang, China (G7, G30, G3003, G3012, G3013, G3014, G3015, G3016, S11, S12, S13, S16, and S22) over the period from 2021 to 2025. The dataset comprises 239 independent observations derived from actual engineering records, which are presented in S1 Table. Each observation corresponds to a specific preventive maintenance project conducted on a defined expressway section within a given year, and can be formally represented as Expressway section × Preventive maintenance treatment × Year.

To account for differences in maintenance technologies, the dataset was divided into three mutually exclusive subsets, namely crack filling (95 samples), surface sealing (75 samples), and overlay (69 samples).

(page 6, lines 194–201)

All preprocessing procedures were applied consistently across the dataset without additional filtering or artificial data augmentation. The complete raw dataset is provided in Supporting Information as S1 Table, and detailed descriptions of all variables are provided in S2 Table, where each record corresponds to the input and output variables used in the predictive models. Following data preprocessing, the

dataset was randomly split into training and testing subsets, with 70% of the data used for training and 30% for testing. The predictive models for maintenance costs were developed using the training dataset, while the test dataset was used to evaluate model performance.

3.1 Data description

(page 15, lines 490–499)

The dataset used in this study is derived from actual maintenance data of Xinjiang expressway pavements, covering three types of preventive maintenance projects: crack filling, surface sealing, and overlay. It includes a total of 239 valid samples, with 95 samples for crack filling, 75 for surface sealing, and 69 for overlay. The core feature variables of the dataset include pavement performance indicators PCI, RQI, RDI, and SRI, all of which comply with the JTG 5210–2018 "Highway Technical Condition Evaluation Standard." Additionally, the dataset contains maintenance quantities, with maintenance cost as the target variable. All data were log-transformed and normalized using Eqs (1) and (2) to eliminate dimensional effects and enhance model training stability.

3.2 Comparative analysis of the results of the prediction models

(page 15, lines 501–511)

The experiment was run on a computer with an AMD Ryzen (TM) 5 5500U CPU @ 2.1 GHz, 64-bit OS, and 64× architecture. To ensure complete experimental reproducibility, a fixed random seed of 42 was set for all Python scripts, model training, and algorithm optimization processes. This study first established three

machine learning models (XGBoost, RF, and BPNN) using Python software to predict preventive maintenance costs for expressways. Then, meta-heuristic algorithms compatible with the models were introduced to optimize the model hyperparameters, resulting in hybrid models (FOA-XGBoost, HOA-RF, and PSO-BPNN). This approach, which combines intelligent optimization algorithms with machine learning models, demonstrated superior predictive accuracy compared to the individual models. The summary of the evaluation metrics for the predictive performance of all six models is shown in Table 5.

Comment 2: Methodological transparency

Important methodological details are insufficiently described, making it difficult to assess how the analyses were conducted and how results were derived.

Response 2:

We fully agree that methodological transparency is essential for clearly understanding how the analyses were conducted and how the results were derived.

In response to this comment, we have improved the description of the model development and optimization process in the Materials and methods section. In particular, Section 2.4 Hybrid optimization algorithms and hyperparameter settings has been substantially revised and expanded to provide a more explicit description of the optimization algorithms, fitness function design, and hyperparameter configuration strategy.

These additions enhance the clarity of the methodological framework and improve the reproducibility of the modeling process. The revised content can be found in Section 2.4 (pages 9–12, lines 270–374) of the manuscript.

Comment 3: Statistical rigor

The statistical analysis relies mainly on descriptive comparisons. Justification of statistical methods, assessment of assumptions, and reporting of uncertainty (e.g., variability, confidence intervals, or effect sizes) are limited.

Response 3:

We appreciate this important comment regarding statistical rigor. We agree that stronger statistical justification and uncertainty quantification are necessary. Specifically, we enhanced the statistical framework by introducing nested cross-validation, reporting mean ± standard deviation and 95% confidence intervals, and applying the Wilcoxon signed-rank test with effect size measures.

The corresponding revisions have been incorporated into the following sections of the manuscript:

Section 2.5.1 Evaluation metrics *(page 12, lines 379–386);*

Section 2.5.2 Nested cross-validation *(page 13, lines 419–423);*

Section 2.6 Statistical significance testing *(page 14, lines 424–432);*

Section 3.3 Wilcoxon signed-rank test results of predictive models *(pages 17–18, lines 541–576).*

Given the extent of these revisions, the full updated text is not reproduced here for brevity.

Comment 4: Strength of conclusions

Some conclusions appear stronger than supported by the presented evidence and should be more cautiously framed.

Response 4:

We thank the reviewer for this comment. We fully agree that some of the original conclusions may have been expressed with a level of certainty exceeding the direct support provided by the current dataset and experimental scope.

In response to this comment, we have carefully revised the manuscript to ensure that the key conclusions are more cautiously framed and more appropriately aligned with the evidence presented in this study. Specifically, we have softened overly strong claims, reduced definitive language, and clarified the scope and limitations of the findings.

The relevant statements have been revised as follows:

1. Original (Abstract):

"Consequently, the developed models can be effectively deployed within

motorway maintenance management departments to achieve efficient resource allocation and refined management of road network maintenance."

Revised:

"Consequently, the developed models may provide useful support for maintenance cost estimation and resource allocation in expressway maintenance management."

2. Original (Case analysis):

"The predictive performance of the FOA-XGBoost, HOA-RF, and PSO-BPNN models has been significantly improved compared to the original base models."

Revised:

"The predictive performance of the FOA-XGBoost, HOA-RF, and PSO-BPNN models shows measurable improvements relative to the original base models for the studied dataset."

3. Original (Conclusion):

"This analysis quantified the contribution degree of input variable to maintenance cost forecasting, and results revealed that maintenance workload emerged as the most critical factor influencing cost variations across all maintenance measures."

Revised:

"These analyses indicate that maintenance workload is an important influencing factor, while different maintenance measures exhibit varying sensitivities to pavement performance indicators."

4. Original (Conclusion):

"In summary, this study has significantly advanced the prediction of preventive maintenance costs for expressways."

Revised:

"In summary, this study contributes to improving the prediction of preventive maintenance costs for expressways."

These revisions ensure that the conclusions are now more cautiously framed.

Comment 5: Data availability and reproducibility

Although supplementary tables are provided, they lack sufficient metadata (e.g., variable definitions and units) and clear linkage to the reported analyses. The underlying datasets required to reproduce the results are not clearly identified or deposited in a public repository, which does not fully comply with the PLOS Data Policy.

Response 5:

This suggestion has helped improve the manuscript. We fully agree that clear data availability, complete metadata, and transparent linkage between the dataset and the reported analyses are essential for reproducibility and compliance with the PLOS Data Policy.

In response to this comment, we have revised the manuscript and Supporting Information to improve data transparency, metadata completeness, and the connection between the dataset and the predictive analyses. Specifically, we have: (1) clarified

the relationship between the data subsets and the corresponding predictive models in Section 2.2 Data sources and preprocessing; (2) supplemented variable definitions, units, and value ranges in Table 2; (3) added a new S2 Table to provide detailed metadata and descriptive information for all variables; and (4) revised S1 Table and added S3 Table to provide the complete raw dataset and fold-specific nested cross-validation results, respectively.

Regarding public data deposition, the complete dataset and associated materials are currently provided in the Supporting Information files. We also attempted to deposit the dataset in figshare, but temporary platform access issues prevented successful upload at this stage. The dataset will be deposited in figshare and made publicly accessible upon publication in accordance with the PLOS Data Policy.

The relevant revised and newly added content is provided below for clarity:

2.2 Data sources and preprocessing

(page 5, lines 153–157)

Separate predictive models were developed for each maintenance category. Accordingly, for modeling purposes, the crack filling dataset is used to develop and evaluate the XGBoost and FOA-XGBoost models, the surface sealing dataset is used to develop and evaluate the RF and HOA-RF models, and the overlay dataset is used to develop and evaluate the BPNN and PSO-BPNN models.

Table 2. Description of the dataset for the prediction model.

Variable type	Name	Description	Unit	Value Range
Input	PCI	Pavement Condition Index, reflecting overall pavement damage condition	—	0–100
	RQI	Road Quality Index, reflecting surface roughness derived from the International Roughness Index	—	0–100
	RDI	Rutting Depth Index, reflecting pavement deformation condition	—	0–100
	SRI	Skid Resistance Index, reflecting pavement friction performance	—	0–100
	Maintenance workload	Quantity of maintenance work performed for a given project	m / m²	Project-dependent
Output	Maintenance costs	Total cost of preventive maintenance for a project	CNY (Yuan)	Project-dependent

Supporting information

(page 22, lines 741–750)

S1 Table. Raw dataset of preventive maintenance projects. *The raw data of pavement technical condition indicators, maintenance workload, and costs for multiple expressway sections with preventive maintenance.*

S2 Table. Metadata and descriptive statistics of model variables. *The metadata and descriptive information of all variables included in the raw dataset and predictive models, including definitions, units, value ranges.*

S3 Table. Nested cross-validation results for predictive models. *The 10-fold nested cross-validation outcomes for six predictive models, showing fold-specific performance metrics.*
