# Supplementary material for: Machine learning approaches for predicting preventive maintenance costs of expressways in Xinjiang
Source: PLoS One. 2026 Jun 16;21(6):e0349595. doi: 10.1371/journal.pone.0349595 (PMC13271440; doi:10.1371/journal.pone.0349595)
Supplement: Peer review history

# Peer review: Decision Letter 0

**DOI:** 10.1371/journal.pone.0349595.r001

**Authors:** Shaheer Ansari

11 Feb 2026

PONE-D-25-52655

Machine learning approaches for predicting preventive maintenance costs of expressways in Xinjiang

PLOS One

Dear Dr. Dai,

Thank you for submitting your manuscript to PLOS ONE. After careful consideration, we feel that it has merit but does not fully meet PLOS ONE’s publication criteria as it currently stands. Therefore, we invite you to submit a revised version of the manuscript that addresses the points raised during the review process.

Please submit your revised manuscript by Mar 28 2026 11:59PM. If you will need more time than this to complete your revisions, please reply to this message or contact the journal office at plosone@plos.org. When you're ready to submit your revision, log on to https://www.editorialmanager.com/pone/ and select the 'Submissions Needing Revision' folder to locate your manuscript file.

Please include the following items when submitting your revised manuscript:

- A letter that responds to each point raised by the academic editor and reviewer(s). You should upload this letter as a separate file labeled 'Response to Reviewers'.
- A marked-up copy of your manuscript that highlights changes made to the original version. You should upload this as a separate file labeled 'Revised Manuscript with Track Changes'.
- An unmarked version of your revised paper without tracked changes. You should upload this as a separate file labeled 'Manuscript'.

If you would like to make changes to your financial disclosure, please include your updated statement in your cover letter. Guidelines for resubmitting your figure files are available below the reviewer comments at the end of this letter.

If applicable, we recommend that you deposit your laboratory protocols in protocols.io to enhance the reproducibility of your results. Protocols.io assigns your protocol its own identifier (DOI) so that it can be cited independently in the future. For instructions see: https://journals.plos.org/plosone/s/submission-guidelines#loc-laboratory-protocols. Additionally, PLOS ONE offers an option for publishing peer-reviewed Lab Protocol articles, which describe protocols hosted on protocols.io. Read more information on sharing protocols at https://plos.org/protocols?utm_medium=editorial-email&utm_source=authorletters&utm_campaign=protocols.

We look forward to receiving your revised manuscript.

Kind regards,

Shaheer Ansari

Academic Editor

PLOS One

Journal Requirements:

When submitting your revision, we need you to address these additional requirements.

1.Please ensure that your manuscript meets PLOS ONE's style requirements, including those for file naming. The PLOS ONE style templates can be found at

https://journals.plos.org/plosone/s/file?id=wjVg/PLOSOne_formatting_sample_main_body.pdf and

https://journals.plos.org/plosone/s/file?id=ba62/PLOSOne_formatting_sample_title_authors_affiliations.pdf

2. Please note that PLOS One has specific guidelines on code sharing for submissions in which author-generated code underpins the findings in the manuscript. In these cases, we expect all author-generated code to be made available without restrictions upon publication of the work. Please review our guidelines at https://journals.plos.org/plosone/s/materials-and-software-sharing#loc-sharing-code and ensure that your code is shared in a way that follows best practice and facilitates reproducibility and reuse.

3. We note that the grant information you provided in the ‘Funding Information’ and ‘Financial Disclosure’ sections do not match.

When you resubmit, please ensure that you provide the correct grant numbers for the awards you received for your study in the ‘Funding Information’ section.

4. Thank you for stating the following financial disclosure:

“This research was funded by the Regional Program of the National Natural Science Foundation of China, grant number: 52562045, Xinjiang Transportation Investment Group Supported Project, grant number: EKXFWCG2024040202, Xinjiang Natural Science Foundation, grant number: 2024D01A53, and the Xinjiang Uygur Autonomous Region “Dr. Tian chi” Project.”

Please state what role the funders took in the study.  If the funders had no role, please state: 'The funders had no role in study design, data collection and analysis, decision to publish, or preparation of the manuscript.'

If this statement is not correct you must amend it as needed.

Please include this amended Role of Funder statement in your cover letter; we will change the online submission form on your behalf.

5. If the reviewer comments include a recommendation to cite specific previously published works, please review and evaluate these publications to determine whether they are relevant and should be cited. There is no requirement to cite these works unless the editor has indicated otherwise.

[Note: HTML markup is below. Please do not edit.]

Reviewer's Responses to Questions

**Comments to the Author**

1. Is the manuscript technically sound, and do the data support the conclusions?

The manuscript must describe a technically sound piece of scientific research with data that supports the conclusions. Experiments must have been conducted rigorously, with appropriate controls, replication, and sample sizes. The conclusions must be drawn appropriately based on the data presented.

Reviewer #1: Yes

Reviewer #2: Partly

**********

2. Has the statistical analysis been performed appropriately and rigorously?

Reviewer #1: Yes

Reviewer #2: No

**********

3. Have the authors made all data underlying the findings in their manuscript fully available?

The PLOS Data policy requires authors to make all data underlying the findings described in their manuscript fully available without restriction, with rare exception (please refer to the Data Availability Statement in the manuscript PDF file). The data should be provided as part of the manuscript or its supporting information, or deposited to a public repository. For example, in addition to summary statistics, the data points behind means, medians and variance measures should be available. If there are restrictions on publicly sharing data—e.g. participant privacy or use of data from a third party—those must be specified.

Reviewer #1: Yes

Reviewer #2: No

**********

4. Is the manuscript presented in an intelligible fashion and written in standard English?

PLOS ONE does not copyedit accepted manuscripts, so the language in submitted articles must be clear, correct, and unambiguous. Any typographical or grammatical errors should be corrected at revision, so please note any specific errors here.

Reviewer #1: Yes

Reviewer #2: Yes

**********

5. Review Comments to the Author

Please use the space provided to explain your answers to the questions above. You may also include additional comments for the author, including concerns about dual publication, research ethics, or publication ethics. (Please upload your review as an attachment if it exceeds 20,000 characters)

Reviewer #1: Strong Aspects

Innovative Integration of Meta-Heuristic Algorithms with Machine Learning

The manuscript demonstrates an advanced integration of meta-heuristic algorithms—specifically the Fruit Fly Optimization Algorithm (FOA), Hiking Optimization Algorithm (HOA), and Particle Swarm Optimization (PSO)—with machine learning models (XGBoost, Random Forest, and Backpropagation Neural Network). This hybridization represents a methodological innovation, enhancing the precision of predictive models for preventive maintenance costs. The study contributes to an emerging research area that fuses intelligent optimization with engineering management, reflecting a sophisticated understanding of model tuning challenges and the necessity of parameter adaptivity in complex datasets.

Comprehensive Framework and Robust Methodological Design

The paper provides a thorough methodological framework encompassing data preprocessing, feature engineering, model construction, optimization, and validation. The implementation of K-fold cross-validation strengthens the reliability of the findings, addressing potential biases in small datasets. Moreover, the feature importance analysis using both model-intrinsic and model-agnostic methods (permutation and SHAP) contributes to the interpretability and transparency of the models, aligning with best practices in machine learning for engineering applications.

Empirical Performance and Practical Relevance

The results reveal remarkable model performance, particularly for PSO-BPNN (R² = 0.9277, MAE = 0.0419), indicating excellent predictive capacity. All hybrid models achieved a Mean Absolute Percentage Error (MAPE) below 5%, which is notable in infrastructure cost forecasting. The study also presents strong applicability: the developed models are suitable for deployment in expressway maintenance management systems, offering practical benefits such as improved resource allocation and budget optimization in highway agencies.

Alignment with Sustainable Development Goals (SDGs)

By promoting cost efficiency and systematic maintenance planning, the study aligns with the United Nations’ Sustainable Development Goal 9—Industry, Innovation, and Infrastructure. Its focus on preventive rather than reactive maintenance highlights an awareness of sustainability and long-term infrastructure resilience.

Weak Aspects

Regional Limitation and Restricted Dataset Scope

The dataset is confined to 13 expressways within Xinjiang, limiting the generalizability of results. The models’ strong predictive performance may be region-specific, as construction practices, material costs, and climatic factors vary substantially across China and other global regions. The exclusion of broader or multi-regional datasets restricts the ability to assess the robustness of these hybrid models under diverse operational conditions

PONE-D-25-52655_reviewer

Absence of Time-Series Analysis

Although the manuscript acknowledges the temporal nature of maintenance costs, it does not incorporate time-series modeling or trend-based learning. Preventive maintenance cost evolution is inherently dynamic, influenced by inflation, traffic growth, and material degradation rates. The absence of time-series models such as Long Short-Term Memory (LSTM) networks or autoregressive integrated moving average (ARIMA) reduces the capacity of the study to forecast future cost trajectories.

Potential Overfitting and Lack of External Validation

Despite the use of cross-validation, the high R² values may suggest overfitting, especially given the relatively small sample size. No independent external validation dataset was used, making it difficult to confirm whether the models can generalize effectively to unseen data. Introducing more robust validation methods or conducting transfer learning across different regions would strengthen confidence in the model’s predictive stability.

Limited Discussion of Economic and Policy Implications

While the technical aspects are well elaborated, the manuscript could more explicitly connect predictive findings to policy and managerial implications for highway authorities. For example, how these models could influence annual budgeting, long-term financial planning, or preventive maintenance prioritization is not extensively discussed. Expanding this discussion would enhance the study’s practical significance.

Recommended Changes

1. Expand the Dataset and Validate Across Regions

Future work should include datasets from other provinces or countries to test the adaptability of the hybrid models under different environmental and economic conditions. This would increase the model’s credibility and relevance for broader infrastructure management applications.

2. Introduce Temporal and Econometric Analysis

Integrating time-dependent variables, such as inflation-adjusted costs, maintenance frequency, or environmental degradation rates, would enable dynamic forecasting. Incorporating time-series models (e.g., LSTM, Prophet, or hybrid ARIMA-ML frameworks) could enhance predictive precision in long-term maintenance planning.

3. Strengthen Validation to Mitigate Overfitting

The authors are encouraged to employ more advanced validation schemes such as nested cross-validation or holdout testing using independent datasets. Sensitivity analysis and hyperparameter uncertainty quantification would further support claims of model robustness.

4. Deepen Ethical and Practical Considerations

Although this research involves infrastructure rather than personal data, ethical and practical implications remain relevant—particularly in algorithmic decision-making that influences public resource allocation. A brief reflection on model transparency, explainability, and decision accountability in automated maintenance systems would be beneficial.

5. Expand Discussion on Policy and Economic Integration

The authors should elaborate on how such predictive tools can be institutionalized within existing Pavement Management Systems (PMS) or governmental budget frameworks. Discussing cost–benefit implications or potential policy adoption pathways would elevate the manuscript’s practical contribution to infrastructure governance. Suggested Citations: DOI: 10.1109/ACCESS.2023.3298955

DOI: 10.1109/ACCESS.2023.3310429

DOI: 10.32604/cmc.2022.023884

DOI: 10.1109/ACCESS.2023.3298955

DOI: 10.3390/biomimetics8030321

Reviewer #2: The manuscript addresses an important topic related to pavement maintenance strategies and presents a substantial dataset comparing different treatment options. The topic is relevant to infrastructure management and has the potential to contribute to practice-oriented decision support. However, several aspects of the study require clarification and strengthening to ensure technical rigor, statistical robustness, and compliance with data availability requirements.

1. Study design and data description

The manuscript does not clearly explain what each observation represents (e.g., spatial unit, time period, or scenario). Key information on data structure, sample size, and replication is missing, limiting the interpretability of the results.

2. Methodological transparency

Important methodological details are insufficiently described, making it difficult to assess how the analyses were conducted and how results were derived.

3. Statistical rigor

The statistical analysis relies mainly on descriptive comparisons. Justification of statistical methods, assessment of assumptions, and reporting of uncertainty (e.g., variability, confidence intervals, or effect sizes) are limited.

4. Strength of conclusions

Some conclusions appear stronger than supported by the presented evidence and should be more cautiously framed.

5. Data availability and reproducibility

Although supplementary tables are provided, they lack sufficient metadata (e.g., variable definitions and units) and clear linkage to the reported analyses. The underlying datasets required to reproduce the results are not clearly identified or deposited in a public repository, which does not fully comply with the PLOS Data Policy.

Overall recommendation

Major revision is required to improve clarity, analytical rigor, and data transparency.

**********

6. PLOS authors have the option to publish the peer review history of their article (what does this mean?). If published, this will include your full peer review and any attached files.

If you choose “no”, your identity will remain anonymous but your review may still be made public.

**Do you want your identity to be public for this peer review?**   For information about this choice, including consent withdrawal, please see our Privacy Policy.

Reviewer #1: No

Reviewer #2: No

**********

[NOTE: If reviewer comments were submitted as an attachment file, they will be attached to this email and accessible via the submission site. Please log into your account, locate the manuscript record, and check for the action link "View Attachments". If this link does not appear, there are no attachment files.]

To ensure your figures meet our technical requirements, please review our figure guidelines: https://journals.plos.org/plosone/s/figures

You may also use PLOS’s free figure tool, NAAS, to help you prepare publication quality figures: https://journals.plos.org/plosone/s/figures#loc-tools-for-figure-preparation.

NAAS will assess whether your figures meet our technical requirements by comparing each figure against our figure specifications.

# Author comment: Author response to Decision Letter 1

**DOI:** 10.1371/journal.pone.0349595.r002

27 Mar 2026

Response to the Reviewer’s Comments

Manuscript number: PONE-D-25-52655

Title: Machine learning approaches for predicting preventive maintenance costs of expressways in Xinjiang

Dear Editors and Reviewers,

We appreciate the opportunity to revise our manuscript titled “Machine learning approaches for predicting preventive maintenance costs of expressways in Xinjiang” and are grateful for the insightful comments provided by the reviewers. Those comments are all valuable and very helpful for revising and improving our paper, and have greatly improved the quality of our work. We have carefully considered all comments and revised the manuscript accordingly. Detailed responses to the reviewers’ comments are provided in the response letter, and the corresponding revisions have been clearly marked in the revised manuscript.

In addition, we have addressed the specific requirements raised by the journal, as detailed below.

1. Code sharing compliance

We have reviewed the PLOS ONE guidelines on code sharing and understand the journal’s requirement that all author-generated code underpinning the findings of the manuscript should be made available without restrictions upon publication of the work. We confirm that all author-generated code underpinning the findings of this study will be made publicly available upon publication of the manuscript.

2. Funding information

We sincerely apologize for the inconsistency between the grant information previously provided in the “Funding Information” and “Financial Disclosure” sections. We have carefully checked and corrected the funding information. This research was supported only by the following two projects:

Xinjiang Transportation Investment (Group) Co., Ltd. Supported Project (Grant No.: EKXFWCG2024040202);

Xinjiang Natural Science Foundation Project (Grant No.: 2024D01A53).

3. Role of funders

The roles of the funders in this study are as follows: Xinjiang Transportation Investment (Group) Co., Ltd. (Grant No.: EKXFWCG2024040202) participated in the study design, provided guidance on data collection methods, and supported data collection and experimental operations financially. Xinjiang Natural Science Foundation Project (Grant No.: 2024D01A53) provided financial support for manuscript revision and offered general suggestions for improvement of the manuscript.

4. Reviewer-suggested references

We have carefully reviewed the references suggested by the reviewers and evaluated their relevance to our study. Relevant works have been considered and incorporated where appropriate in the revised manuscript.

We have carefully followed the journal’s revision requirements and sincerely hope that the revised manuscript and response letter meet the standards for publication in PLOS ONE.

Thank you very much for your time, consideration, and valuable guidance.

Sincerely,

The Authors

Reviewer #1:

Comment 1: Expand the Dataset and Validate Across Regions

Future work should include datasets from other provinces or countries to test the adaptability of the hybrid models under different environmental and economic conditions. This would increase the model’s credibility and relevance for broader infrastructure management applications.

Response 1:

We appreciate this important suggestion. We agree that cross-regional validation would further enhance the generalizability of the proposed models. Due to the limited availability of consistent and high-resolution maintenance cost data across regions, such validation could not be incorporated in the current study. To partially address this concern, we explicitly outlined cross-regional validation as a key direction for future work.

We have added the following statement in the Conclusion section (page 21, lines 700–704):

“Further integration of expressway maintenance data from regions with more diverse climatic conditions and socioeconomic backgrounds, such as southern China and other countries worldwide, would enable more robust validation and refinement of the hybrid model proposed in this study, thereby enhancing its generalizability and practical utility for infrastructure management in broader contexts.”

Comment 2: Introduce Temporal and Econometric Analysis

Integrating time-dependent variables, such as inflation-adjusted costs, maintenance frequency, or environmental degradation rates, would enable dynamic forecasting. Incorporating time-series models (e.g., LSTM, Prophet, or hybrid ARIMA-ML frameworks) could enhance predictive precision in long-term maintenance planning.

Response 2:

We agree that incorporating temporal dynamics would improve long-term predictive capability. However, in the current study, the available dataset mainly consists of short-term panel data (2021–2025), which is not sufficient to support the training and validation of complex time-series models. In addition, the primary objective of this study is to perform a cross-sectional comparison of feature engineering and machine learning optimization under different preventive maintenance strategies. We have added a discussion on the integration of temporal models and macroeconomic variables in future work.

To address this important comment, we have added a corresponding discussion in the Conclusion section of the revised manuscript (page 21, lines 704–707):

“Simultaneously, collecting long-term monitoring data and incorporating macroeconomic variables, together with time-series models such as LSTM or ARIMA-based hybrid approaches, would enable dynamic maintenance cost prediction over longer life cycles.”

Comment 3: Strengthen Validation to Mitigate Overfitting

The authors are encouraged to employ more advanced validation schemes such as nested cross-validation or holdout testing using independent datasets. Sensitivity analysis and hyperparameter uncertainty quantification would further support claims of model robustness.

Response 3:

We fully agree that rigorous validation and robustness analysis are essential for reducing overfitting risk and improving the reliability of predictive models.

In response to this comment, we have substantially strengthened both the validation framework and the robustness analysis in the revised manuscript. Specifically, we replaced the original 5-fold cross-validation with a 10-fold nested cross-validation scheme, and incorporated single-factor sensitivity analysis to further evaluate model robustness and interpretability. Corresponding revisions were made in both the Materials and methods and Case study sections.

More specifically, the relevant revisions have been incorporated into the following sections of the revised manuscript:

Section 2.5 Model evaluation (page 12, lines 375–378), where the evaluation framework has been clarified;

Section 2.5.2 Nested cross-validation (page 13, lines 396–423), where the nested cross-validation procedure and confidence interval estimation have been added;

Section 2.7 Influencing factor analysis and sensitivity analysis (page 15, lines 478–488), where the rationale and procedure of sensitivity analysis are described;

Section 3.4 Cross-validation experimental results (pages 18–19, lines 577–611), where the validation results under the revised framework are reported;

Section 3.5 Results of influencing factor and sensitivity analyses (pages 19–20, lines 612–649), where the robustness and interpretability results are presented.

Comment 4: Deepen Ethical and Practical Considerations

Although this research involves infrastructure rather than personal data, ethical and practical implications remain relevant—particularly in algorithmic decision-making that influences public resource allocation. A brief reflection on model transparency, explainability, and decision accountability in automated maintenance systems would be beneficial.

Response 4:

We fully agree with the reviewer, although this study does not involve personal data, ethical and practical considerations remain highly relevant, particularly in the context of algorithmic decision-making for public resource allocation.

In response to this important concern, we have added a dedicated discussion in the Conclusion section of the revised manuscript (page 21, lines 720–727), addressing issues related to model transparency, interpretability, and decision accountability in practical applications. The added text reads:

“In practical applications, additional considerations are also necessary. When applied to public resource allocation, issues related to transparency, interpretability, and decision accountability should be carefully addressed. The integration of explainable artificial intelligence (XAI) techniques can help clarify the contribution of input features and improve model interpretability. At the same time, the proposed models are intended to serve as decision-support tools rather than fully automated decision-making systems, and human oversight remains essential to ensure responsible and accountable use of maintenance resources.”

Comment 5: Expand Discussion on Policy and Economic Integration

The authors should elaborate on how such predictive tools can be institutionalized within existing Pavement Management Systems (PMS) or governmental budget frameworks. Discussing cost–benefit implications or potential policy adoption pathways would elevate the manuscript’s practical contribution to infrastructure governance.

Response 5:

We sincerely thank the reviewer for this valuable suggestion. We agree that further elaboration on policy integration and practical implementation would enhance the real-world relevance and applicability of the proposed models.

To address this comment, we have expanded the discussion in the Conclusion section of the revised manuscript (page 21, lines 728–736), where we describe how the proposed predictive framework can be integrated into existing Pavement Management Systems (PMS) and its potential role in supporting budget planning and decision-making processes. The added text reads:

“From an application perspective, the proposed hybrid models can be integrated into existing Pavement Management Systems (PMS) as a cost prediction module. Once maintenance needs are identified within the PMS, the appropriate model can be selected to generate cost estimates based on key pavement performance indicators. This data-driven approach can support preliminary budget planning, facilitate a transition from reactive to preventive maintenance strategies, and improve cost-effectiveness over the entire lifecycle. Furthermore, such integration provides a pathway for embedding data-driven cost estimation into existing decision-support workflows within infrastructure management agencies.”

We sincerely thank the reviewer for recommending these high-quality and relevant references. We have carefully reviewed these studies and incorporated them into the revised manuscript in appropriate sections to strengthen the literature review and methodological grounding of the study.

Specifically, Refs. [27] and [28] have been cited in the Introduction section (page 3, lines 63–65) in support of the following statement:

“Consequently, a growing body of research has focused on integrating metaheuristic algorithms with machine learning models to develop more effective hybrid models [27,28].”

In addition, Ref. [43] has been added in the Materials and methods section (page 14, line 429), Wilcoxon signed-rank test to support the statistical comparison between the optimized hybrid models and their corresponding baseline models. The revised text reads:

“To evaluate whether the performance differences between the optimized hybrid models and their baseline counterparts are statistically significant, the Wilcoxon signed-rank test was conducted based on cross-validation results, where key performance metrics (e.g., MAE and RMSE) obtained from each fold were treated as paired samples for model comparison. Statistical significance was determined at a predefined level of α = 0.05 [43].”

The newly added references have also been marked in the reference list for ease of checking.

Reviewer #2:

Comment 1: Study design and data description

The manuscript does not clearly explain what each observation represents (e.g., spatial unit, time period, or scenario). Key information on data structure, sample size, and replication is missing, limiting the interpretability of the results.

Response 1:

This is an important point. We agree that a clear description of the study design and dataset structure is essential for improving the interpretability and reproducibility of the results.

In response to this comment, we have substantially revised the manuscript to clarify the definition of observations, dataset organization, and sample distribution. Specifically, we now explicitly define each observation as an individual preventive maintenance project represented by “expressway section × maintenance treatment × year”, and we provide detailed information on the dataset structure, total sample size (239 observations), and the sample sizes for each maintenance category.

We have also revised the Data sources and preprocessing section to clearly describe the dataset organization and its role in the modeling framework, and we explicitly state that the complete raw dataset is provided in S1 Table, with consistent preprocessing procedures applied across all samples. In addition, a fixed random seed has been introduced to enhance the reproducibility of the experiments.

The revised and newly added text is provided below for clarity:

2.2 Data sources and preprocessing

(pages 4–5, lines 144–150)

This study collected data from preventive maintenance projects implemented on 13 expressways in Xinjiang, China (G7, G30, G3003, G3012, G3013, G3014, G3015, G3016, S11, S12, S13, S16, and S22) over the period from 2021 to 2025. The dataset comprises 239 independent observations derived from actual engineering records, which are presented in S1 Table. Each observation corresponds to a specific preventive maintenance project conducted on a defined expressway section within a given year, and can be formally represented as Expressway section × Preventive maintenance treatment × Year.

To account for differences in maintenance technologies, the dataset was divided into three mutually exclusive subsets, namely crack filling (95 samples), surface sealing (75 samples), and overlay (69 samples).

(page 6, lines 194–201)

All preprocessing procedures were applied consistently across the dataset without additional filtering or artificial data augmentation. The complete raw dataset is provided in Supporting Information as S1 Table, and detailed descriptions of all variables are provided in S2 Table, where each record corresponds to the input and output variables used in the predictive models. Following data preprocessing, the dataset was randomly split into training and testing subsets, with 70% of the data used for training and 30% for testing. The predictive models for maintenance costs were developed using the training dataset, while the test dataset was used to evaluate model performance.

3.1 Data description

(page 15, lines 490–499)

The dataset used in this study is derived from actual maintenance data of Xinjiang expressway pavements, covering three types of preventive maintenance projects: crack filling, surface sealing, and overlay. It includes a total of 239 valid samples, with 95 samples for crack filling, 75 for surface sealing, and 69 for overlay. The core feature variables of the dataset include pavement performance indicators PCI, RQI, RDI, and SRI, all of which comply with the JTG 5210–2018 “Highway Technical Condition Evaluation Standard.” Additionally, the dataset contains maintenance quantities, with maintenance cost as the target variable. All data were log-transformed and normalized using Eqs (1) and (2) to eliminate dimensional effects and enhance model training stability.

3.2 Comparative analysis of the results of the prediction models

(page 15, lines 501–511)

The experiment was run on a computer with an AMD Ryzen (TM) 5 5500U CPU @ 2.1 GHz, 64-bit OS, and 64× architecture. To ensure complete experimental reproducibility, a fixed random seed of 42 was set for all Python scripts, model training, and algorithm optimization processes. This study first established three machine learning models (XGBo

Attachment Submitted filename: Response to Reviewers.pdf

# Peer review: Decision Letter 1

**DOI:** 10.1371/journal.pone.0349595.r003

**Authors:** Shaheer Ansari

3 May 2026

Machine learning approaches for predicting preventive maintenance costs of expressways in Xinjiang

PONE-D-25-52655R1

Dear Dr. Dai,

We’re pleased to inform you that your manuscript has been judged scientifically suitable for publication and will be formally accepted for publication once it meets all outstanding technical requirements.

Within one week, you’ll receive an e-mail detailing the required amendments. When these have been addressed, you’ll receive a formal acceptance letter and your manuscript will be scheduled for publication.

An invoice will be generated when your article is formally accepted. Please note, if your institution has a publishing partnership with PLOS and your article meets the relevant criteria, all or part of your publication costs will be covered. Please make sure your user information is up-to-date by logging into Editorial Manager at Editorial Manager® and clicking the ‘Update My Information' link at the top of the page. For questions related to billing, please contact billing support.

If your institution or institutions have a press office, please notify them about your upcoming paper to help maximize its impact. If they’ll be preparing press materials, please inform our press team as soon as possible -- no later than 48 hours after receiving the formal acceptance. Your manuscript will remain under strict press embargo until 2 pm Eastern Time on the date of publication. For more information, please contact onepress@plos.org.

Kind regards,

Shaheer Ansari

Academic Editor

PLOS One

# Editor report: Acceptance letter

**DOI:** 10.1371/journal.pone.0349595.r004

**Authors:** Shaheer Ansari

PONE-D-25-52655R1

PLOS One

Dear Dr. Dai,

I'm pleased to inform you that your manuscript has been deemed suitable for publication in PLOS One. Congratulations! Your manuscript is now being handed over to our production team.

At this stage, our production department will prepare your paper for publication. This includes ensuring the following:

* All references, tables, and figures are properly cited

* All relevant supporting information is included in the manuscript submission,

* There are no issues that prevent the paper from being properly typeset

You will receive further instructions from the production team, including instructions on how to review your proof when it is ready. Please keep in mind that we are working through a large volume of accepted articles, so please give us a few days to review your paper and let you know the next and final steps.

Lastly, if your institution or institutions have a press office, please let them know about your upcoming paper now to help maximize its impact. If they'll be preparing press materials, please inform our press team within the next 48 hours. Your manuscript will remain under strict press embargo until 2 pm Eastern Time on the date of publication. For more information, please contact onepress@plos.org.

You will receive an invoice from PLOS for your publication fee after your manuscript has reached the completed accept phase. If you receive an email requesting payment before acceptance or for any other service, this may be a phishing scheme. Learn how to identify phishing emails and protect your accounts at https://explore.plos.org/phishing.

If we can help with anything else, please email us at customercare@plos.org.

Thank you for submitting your work to PLOS ONE and supporting open access.

Kind regards,

PLOS ONE Editorial Office Staff

on behalf of

Dr. Shaheer Ansari

Academic Editor

PLOS One
